# Anti-PD-1 antibody increases NK cell cytotoxicity towards nasopharyngeal carcinoma cells in the context of chemotherapy-induced upregulation of PD-1 and PD-L1

**DOI:** 10.1007/s00262-020-02681-x

**Published:** 2020-07-31

**Authors:** Anna Makowska, Selina Meier, Lian Shen, Pierre Busson, Valentin Baloche, Udo Kontny

**Affiliations:** 1 Division of Pediatric Hematology, Oncology and Stem Cell Transplantation, Medical Faculty, Rhenish-Westphalian Technical University, Pauwelsstraße 30, 52074 Aachen, Germany; 2 CNRS UMR 8126, Gustave Roussy and Université Paris-Sud/Paris-Saclay, Villejuif, France

**Keywords:** Nasopharyngeal carcinoma, Natural killer cells, Programmed cell death ligand 1, Chemotherapy, Nuclear factor kappa b, Interferon beta

## Abstract

**Background:**

Nasopharyngeal carcinoma (NPC) is a highly malignant epithelial cancer linked to Epstein–Barr virus (EBV) infection. Tumors are characterized by a lymphomononuclear infiltrate and the number of natural killer (NK) cells in tumors appears to be of prognostic significance. Standard treatment for NPC in adolescents and young adults consists of induction chemotherapy followed by radiochemotherapy. Though survival rates are above 80%, the majority of patients suffer from long-term side-effects, mainly related to radiotherapy. The addition of immunotherapy to induction chemotherapy could improve tumor response.

**Methods:**

We have investigated the killing of NPC cells by NK cells in the context of chemotherapy, using a panel of three nasopharyngeal carcinoma cell lines and a patient-derived xenograft. Cytotoxicity was measured using the calcein-release assay, while the contribution of different checkpoints and signaling pathways to killing was studied by siRNA-mediated gene silencing and chemical inhibitors.

**Results:**

Chemotherapeutics cisplatin, 5-fluorouracil and gemcitabine sensitized NPC cells to killing by NK cells. Chemotherapeutics led to upregulation of PD-1 in NK cells and PD-L1 in NPC cells via NF-κB. Inhibition of the PD-L1/PD-1 checkpoint by an anti-PD-1 antibody or siRNA increased NK-cell cytotoxicity towards NPC cells.

**Conclusion:**

The addition of an anti-PD-1 antibody to chemotherapy in patients with NPC could increase the efficacy of induction chemotherapy. If confirmed in a clinical trial, more efficient induction therapy could allow the dose of radiotherapy to be reduced and thereby diminish severe late effects of such therapy.

**Electronic supplementary material:**

The online version of this article (10.1007/s00262-020-02681-x) contains supplementary material, which is available to authorized users.

## Introduction

Nasopharyngeal carcinoma (NPC) is a tumor arising from the epithelial lining of the posterior nasopharynx [1]. Its incidence varies worldwide, with a high incidence in Southeast Asia, particularly Southern China. The occurrence of NPC is associated with Epstein–Barr virus (EBV) infection, and dysregulation of immune function has been hypothesized to play an important role in the development of NPC [2].

Radiochemotherapy is the main treatment modality in patients with NPC, leading to event-free survival (EFS) rates > 80% for patients with locally advanced tumors [3]. Standard radiation dosages used are around 70 Gy and are associated with severe long-term sequelae such as xerostomia, endocrinopathies or secondary neoplasms [4]. Induction chemotherapy has been introduced as a standard in treatment protocols for younger patients allowing lower radiation dosages to decrease the risk of severe late effects [5–7]. The NPC 2003 protocol of the German Society of Pediatric Oncology and Hematology (GPOH) successfully decreased the radiation dosage to the primary tumor from 59.4 to 54 Gy in patients with a complete response after 3 blocks of induction chemotherapy with 5-fluoruracil and cisplatin. EFS and overall survival (OS) rates were 92.4% and 97.1%, respectively [5].

There is evidence that NPC is susceptible to immunotherapeutic approaches, as the adoptive transfer of EBV-specific T cells or blockade of the PD-L1/PD-1 checkpoint has led to tumor responses and disease control in some patients with refractory disease [8–10]. Maintenance therapy with interferon-β (IFNβ) was introduced in the last two GPOH-NPC-trials and resulted in EFS and OS rates > 90% [5,11]. IFNβ has been shown to activate NK cells from NPC patients and to increase their ability to kill NPC cells in vitro [12]. NK cells are one of the key effectors of the host anti-tumor immune response [13]. A role for NK cells in NPC has also been suggested by the observations that patients with NPC often have dysfunctional NK cells at diagnosis and that the degree of NK-cell infiltration of NPC tumors positively correlates with prognosis [14,15]. We have previously shown that IFNβ induces PD-L1 expression in NPC cells and PD-1 expression in NK cells, and that blocking of the PD-L1/PD-1 checkpoint augments the cytotoxic effect of NK cells on NPC cells [16]. Chemotherapeutics are known to upregulate PD-L1 in cancer cells, and the addition of an anti-PD1 antibody to platin-containing induction chemotherapy has proved to significantly increase the response rate in patients with non-small cell lung cancer [17,18]. As neoadjuvant chemotherapy is nowadays increasingly used also in adult patients with NPC [19], we decided to investigate the effect of chemotherapeutics on the killing of NPC cells by NK cells.

## Materials and methods

### Cell lines

The NPC cell lines C666-1 [44], CNE-2 [45] and TW01 [46] were used. Cell lines were maintained in RPMI1640 medium (Gibco, Paisley, UK) supplemented with 10% fetal bovine serum (Gibco, Paisley, UK), 100 U/ml penicillin and 100 mg/ml streptomycin (Gibco; NY, USA). Cells were cultured in a humidified incubator with 95% air and 5% CO_2_ at 37 °C.

### Patient-derived xenograft

The xenograft C17 was established from a patient with an EBV-positive metastatic NPC by Prof. Pierre Busson, Paris in nude mice [20]. For the experiments described below, single cells suspensions were derived from freshly isolated C17 tumor fragments by collagenase cell dispersion and cultured as described before [12,16].

### Animal studies

Swiss nude mice were bred in the animal facility at Gustave Roussy and housed in pathogen-free conditions in filter cap cages holding a maximum of 5 animals with irradiated aspen chip bedding and cotton fiber nesting material. They were maintained on a 12/12 light/dark cycle, with ad libitum UV-treated water and RM1 rodent diet. Typically, xenografts were performed on 6–8 female mice by subcutaneous introduction of tumor fragments (about 200 mg) under general anesthesia. They were sacrificed when the total tumor volume reached 1700 mm^3^. The animals were monitored for signs of pain, such as immobility or restlessness, reduction of drinking and food intake. The persistence of abnormal behaviors led to the euthanasia of animals presumed to be suffering. Prior to tumor collection, mice were sacrificed by cervical dislocation. Otherwise, mice were euthanatized by carbon dioxide asphyxiation.

### Isolation of primary human NK cells

Human peripheral blood mononuclear cells (PBMC) were purified from buffy coats of 5 healthy donors using Ficoll-Hypaque (Biochrom; Berlin, Germany) density gradient centrifugation. Informed consent was obtained from all donors. Positive magnetic selection of CD56^+^ cells from PBMCs was performed according to the manufacturer’s instructions (Miltenyi; Bergisch Gladbach, Germany). Purified NK cells were held in RPMI1640 medium supplemented with 10% FCS and 100 U/ml penicillin and 100 mg/ml streptomycin (Gibco; NY, USA) and immediately used for experiments. Remaining cells were frozen at − 80 °C and used for subsequent experiments after thawing.

### Reagents

Chemotherapeutics used for this study were cisplatin (Teva; Ulm, Germany), 5-fluorouracil (Medac; Hamburg, Germany) and gemcitabine (Hexal; Holzkirchen, Germany). Methylthiazolyldiphenyl-tetrazolium bromide (MTT) was obtained from Sigma Aldrich (St. Louis; MO, USA), human recombinant interferon beta from R&D System (New York; NY, USA), the PD-1 inhibitor nivolumab from Bristol-Myers (Anagni, Italy). Calcein-AM was purchased from Thermo Fisher (Eugene, USA), soluble, recombinant human TRAIL from Enzo Life Science (Paris, France). Primary mouse monoclonal antibody against PD-1, clone 913429 was obtained from R&D System (Wiesbaden, Germany), anti-human B7-H1/PD-L1 monoclonal antibody, clone 130021 from R&D System (Minneapolis, USA). Knock-down experiments were performed using PD-L1-siRNA (Dharmacon; Freiburg, Germany, Cat. Nr. L-015836-01-0010), RELA-siRNA for NF-κB (Dharmacon; Cat. Nr. L-003533-00-0005) and scrambled RNA (Dharmacon; Cat. Nr. D-001810-20-0005). Antibodies used for immunoblotting were mouse anti-human *β*-actin (Cell Signaling; Danvers, MA, USA), mouse anti-human-PD-1 (R&D System), mouse anti-human-PD-L1 (Clone # 130021; R&D System) and mouse anti-human-RELA/NF-κB p65 (Clone #532301; R&D System). The goat anti-mouse IgG secondary antibody was purchased from Santa Cruz Biotechnology (Heidelberg, Germany). NF-κB inhibitor—BMS-345541 was obtained from Sigma Aldrich.

### Cell viability assay

The MTT assay was used to determine the effect of cisplatin (0 to 80 µg/ml), 5-fluorouracil (0–64 µg/ml) and gemcitabine (0–80 µg/ml) on cell viability. Cells were seeded into 96-well plates at a density of 2.500 cells/well within 200 µl of growth medium. After 24 h of culture, cells were treated with different concentrations of chemotherapeutics and incubated for 24, 48 and 72 h. At the end of the incubation periods MTT was added and optical density measured as described before [21].

### Cell cycle analysis

Propidium-iodide staining of nuclei was used to determine the effect of chemotherapeutics on cell cycle distribution as well as apoptosis by measurement of sub-G1 DNA content. NPC cells (about 70% confluent) were incubated with cisplatin (0–80 µg/ml), 5-fluorouracil (0–64 µg/ml) and gemcitabine (0–80 µg/ml) or medium up to 72 h. Cells were then processed as described previously [16,21]. Three independent experiments were performed for each assay.

### Determination of chemotherapy concentrations

Preliminary experiments were performed to identify the chemotherapy dosages for the main experiments. A cisplatin concentration of 2.5 µg/ml, 5-fluorouracil concentration of 32 µg/ml and gemcitabine concentration of 10 µg/ml were identified as dosages significantly decreasing cell survival and inducing apoptosis in NPC cell lines and were thus chosen for further experiments (Suppl. Figure 1A, 1B). The concentrations selected were all within the range of serum concentrations measured in patients treated with the respective agents. In humans, serum concentrations of up to 4.0 µg/ml for cisplatin [22], 48.41 µg/ml for 5-fluorouracil [23] and 10.27 µg/ml for gemcitabine [22] are achieved at therapeutic dosages in patients with solid tumors. In addition, calcein-release assays were performed for all cell lines. Each cell line was treated for 24 h with the various chemotherapeutic agents at the selected concentrations (Suppl. Figure 1C).

### Flow cytometry

NPC and NK cells untreated or treated with 2.5 µg/ml cisplatin, 32 µg/ml 5-fluorouracil or 10 µg/ml gemcitabine for 24 h were suspended at a density of 1 × 10^6^ cells in 500 µl of medium and incubated with 5 µl of mouse anti-human PD-L1 or PD-1-antibody for 1 h on ice. For detection of NF-κB expression, cells were pelleted, resuspended in 1 ml Cytofix/Cytoperm™ Fixation and Permeabilization Solution (BD Pharmingen; San Diego, USA) and incubated for 20 min on ice. The suspension was then centrifuged, and the pellet washed with Perm/Wash™ Buffer (BD Pharmingen). Labeling was performed by adding 500 μl PBS and 5 µl of mouse anti-human NF-κB-antibody to the cells. The samples were incubated for 1 h on ice. After washing in PBS three times (5 min each), APC-conjugated goat-anti-mouse antibody (1:200) was added to the cell suspensions, which were then incubated for 1 h on ice. Subsequent to rinsing in PBS, samples were analyzed by flow cytometry. Data were analyzed by the FlowJo software (FlowJo). Three independent experiments were performed for each assay.

### Calcein release assay

The fluorescence-based calcein-AM release assay was used to determine the effect of NK cell-induced cytotoxicity. The assay was performed as reported before [12,16]. Briefly, after treatment NPC cells were washed and resuspended in 15 µM calcein-AM for 30 min at 37 °C. Cells were then co-incubated with NK cells at an effector to target (E:T) ratio of 6:1 for 4 h at 37 °C or used as controls, 4% Triton (Merck; Darmstadt, Germany) was added to ensure maximum calcein release in controls while spontaneous calcein release was measured in NPC cells not co-incubated with NK cells (Suppl. Figure 2). After co-incubation, relative fluorescence units (RFU) were measured from cell free supernatant. The percentage of specific lysis was calculated as follows: [(RFU value in respective treatment − RFU value in control (spontaneous release))/(RFU value Triton (maximum release) − RFU value in control (spontaneous release)) × 100].

### Immunoblot

Immunoblotting was performed as described before [21]. Briefly, cells were washed and lysed in buffer containing 50 mM Tris–HCl (pH 7.4), 1% NP-40, 0.5% Na-deoxycholate, 0.1% SDS, 150 mM NaCl, 2 mM EDTA, 50 mM NaF and protease and phosphatase inhibitors. Cellular proteins from total cell lysates (20 µg/sample) were separated by sodium dodecyl sulfate-polyacrylamide gel electrophoresis and transferred onto a nitrocellulose membrane. The membranes were probed using immunoblot analyses with mAb to human PD-1 (1:1000), PD-L1 (1:500) and NF-κB (1:1000), followed by incubation with the goat anti-rabbit IgG-antibody for 1 h at room temperature. Equal protein loading was confirmed by reprobing filters with a monoclonal antibody against *β*-actin. Immunoreactive bands were detected using enhanced chemiluminescence and visualized by autoradiography.

### Transfection of siRNA

NPC cells were seeded at 10^5^ cells/well in 24-well plates. The following day, when cells had reached about 80% confluency, the culture medium was aspirated and cells were washed with PBS before being transfected with lipofectamine (Invitrogen; Carlsbad, CA, USA) of siRNA against PD-L1, NF-κB or scrambled siRNA. After 16 h the transfection mix was replaced with normal growth medium and the cells were treated with 2.5 µg/ml cisplatin, 32 µg/ml 5-fluorouracil or 10 µg/ml gemcitabine for 24 h. Transfection efficiency was monitored by measuring the surface expression of PD-L1 and NF-κB using flow cytometry and immunoblotting. Three independent experiments were performed for each assay.

NK cells were nucleofected with siRNA against NF-κB or scrambled siRNA according to the protocols supplied by Amaxa (Köln, Germany). In brief, 5 × 10^6^ NK cells per nucleofection were pelleted, washed once in sterile PBS and resuspended in 100 μl of NK-cell nucleofector solution (Amaxa). The indicated concentrations of siRNAs were added to the cell suspensions, mixed gently and transferred immediately to the relevant cuvettes. The cuvettes were placed in the Amaxa Nucleofector^®^ Device and pulsed using the appropriate program. The cells were removed from the cuvette and incubated in RPMI 1640 containing 10% fetal calf serum without antibiotics. After transfection, the cells were treated as transfected NPC cells above. Transfection efficiency was monitored by measuring surface expression of PD-1 and NF-κB using flow cytometry and immunoblotting. Three independent experiments were performed for each assay.

### Statistical analysis

Experimental results were reported as a mean of at least three independent experiments conducted in quintuplicates for cell viability assays and triplicates for flow cytometric analyses. Data in bar graphs are presented as means ± S.E. Student’s *t* test was used to compare two sets of data, taking *P* < 0.05 as statistically significant.

## Results

### Chemotherapeutic agents sensitize NPC cells to killing by NK cells

**Fig. 1:**
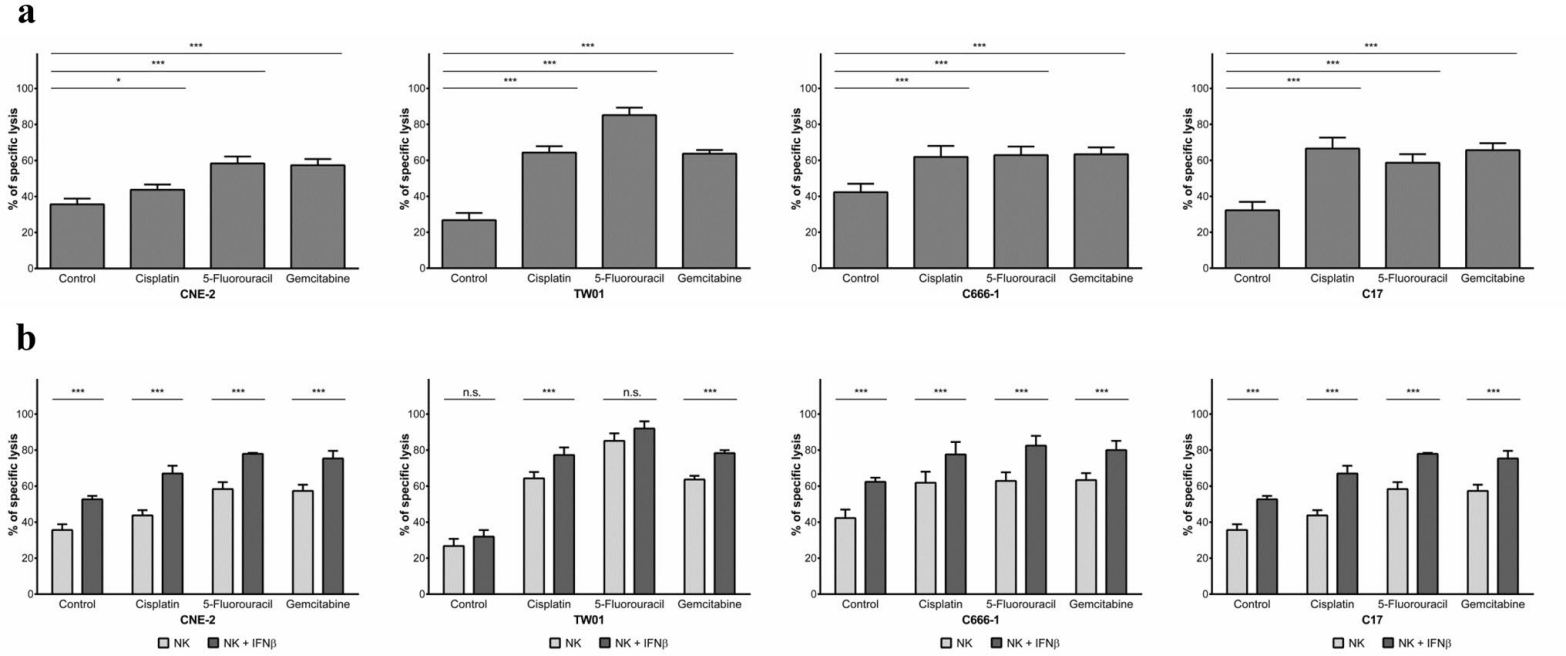
Chemotherapeutics sensitize NPC cells to killing by NK cells. NPC cells were incubated for 24 h with either cisplatin (2.5 µg/ml), 5-fluorouracil (32 µg/ml) or gemcitabine (10 µg/ml). Target cells were labeled with calcein, plated in a 96-well plate and incubated with NK cells for 4 h at an E:T ratio of 6:1. **a** Lysis of target cells was determined by measurement of calcein in collected supernatants by an ELISA reader. Data are presented as means ± S.E.M. Asterisks indicate statistically significant differences between all cell lines in one ratio-group (two-way ANOVA; *P < 0.05; **P < 0.01; ***P < 0.001). **b** NK cells were pre-incubated or not with IFNβ (1000 U/ml) for 24 h and then co-incubated with NPC cells as above

Anticancer chemotherapeutic drugs, as well as NK cells, induce apoptosis and may therefore share a common intracellular signaling pathway leading to cell death. Indeed, several recent reports have demonstrated that several chemotherapeutic drugs augment NK cell-induced killing of different types of carcinoma cells [24]. To assess the influence of chemotherapy on the cytotoxic effect of NK cells against NPC cells, we pre-incubated NPC cells with the anticancer drugs cisplatin, 5-fluorouracil or gemcitabine, which are commonly used in the treatment of NPC patients. The concentrations used were determined in preliminary experiments and decreased cell viability to between 10 and 40% after 24 h (Suppl. Figure 1A); these concentrations lie within the range required to achieve a therapeutic effect in cancer patients [28,29]. In our experiments, NPC cells were incubated for 24 h with anticancer drugs, then labeled with calcein, and subsequently co-incubated with NK cells. After co-incubation, the concentration of calcein in the supernatant was measured as a marker for NPC cytotoxicity. Treatment of NPC cells with NK cells for 4 h at an E:T ratio of 6:1 induced considerable killing in all NPC cell lines, ranging from 26.72% for cell line TW01 to 42.28% for C666-1. Pre-treatment of NPC cells with chemotherapeutics increased their killing by NK cells further up to around 80% (Fig. 1a).

Having previously demonstrated that activation of NK cells with IFNβ significantly increased their killing of NPC cells [12,16], we next asked the question, whether killing of NPC cells exposed to chemotherapeutic agents was further augmented when NK cells were activated. In this experiment, NK cells were exposed to 1000 U/ml IFNβ for 24 h and then co-incubated with NPC cells pre-incubated with the anticancer drugs as above. IFNβ significantly increased NK cytotoxicity towards NPC cells pretreated with chemotherapeutic agents by an average of 14.38% in all cell lines when compared to extermination by non-activated NK cells (Fig. 1b). IFNβ had no significant effect on cell viability or apoptosis of treated NK cells (Suppl. Figure 3).

### Chemotherapeutic agents induce upregulation of PD-L1 in NPC cells

Anticancer agents are able to alter the expression of genes influencing the interaction of cancer cells with the microenvironment and the immune system. Recent studies have shown that chemotherapeutic agents induce the expression of the negative checkpoint ligand PD-L1 in cancer cells and that blocking of the PD-L1/PD-1 checkpoint increases the responsiveness of NSCLC to chemotherapy in patients [18].

**Fig. 2:**
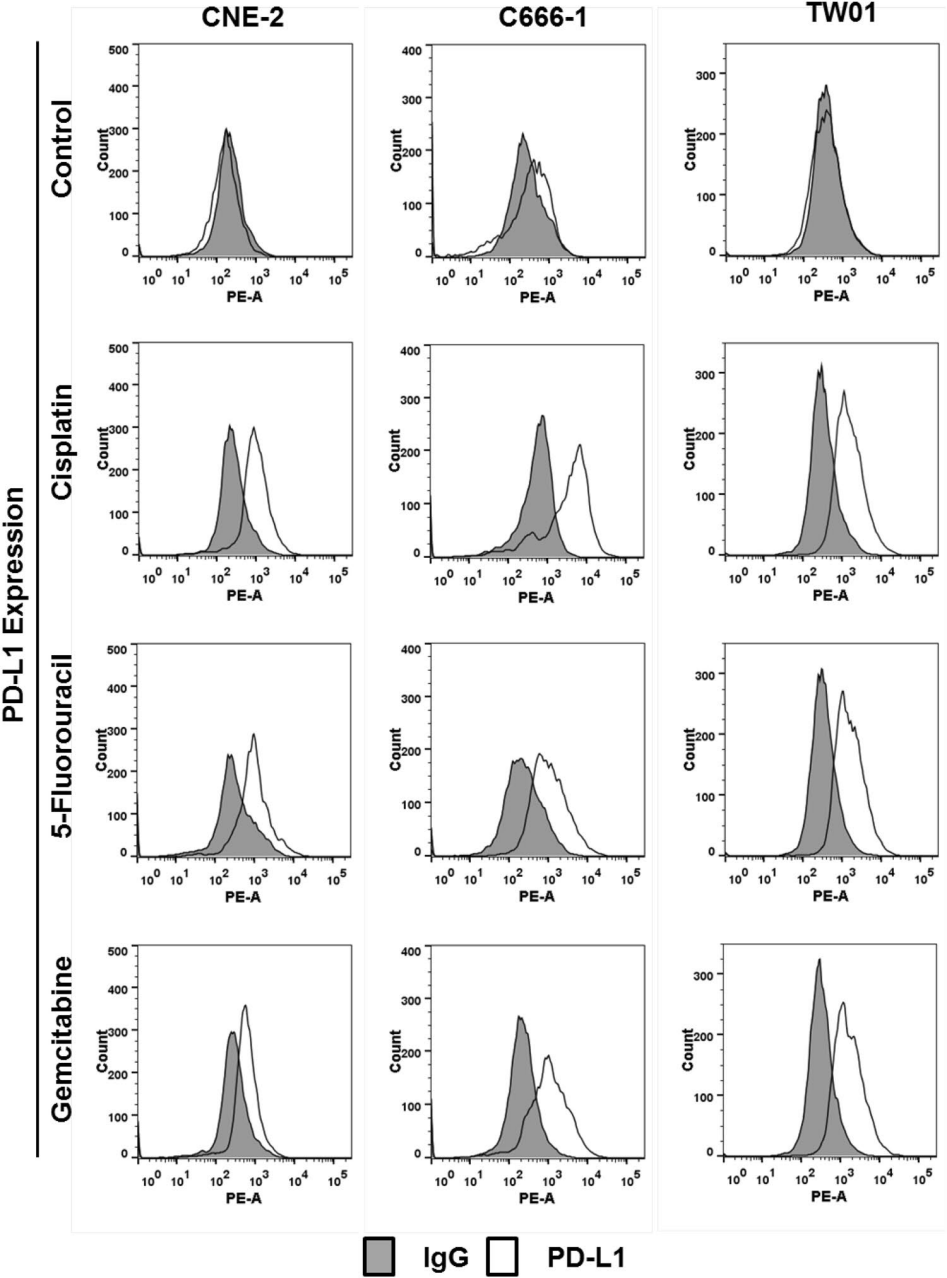
Chemotherapeutics induce surface expression of PD-L1 in NPC cells. PD-L1 surface expression was measured by flow cytometry 24 h after incubation of NPC cells with cisplatin (2.5 µg/ml), 5-fluorouracil (32 µg/ml) and gemcitabine (10 µg/ml), respectively

To determine whether PD-L1 expression in NPC cells is modulated by chemotherapy, NPC cells were incubated with cisplatin, 5-fluorouracil and gemcitabine for 24 h, and PD-L1 expression was then determined by flow cytometry. All three chemotherapeutic agents significantly induced PD-L1 surface expression in each of the three NPC cell lines studied. No surface expression of PD-L1 was observed in untreated NPC cells (Fig. 2).

### Inhibition of PD-1 increases killing of NPC cells by activated NK cells.

**Fig. 3:**
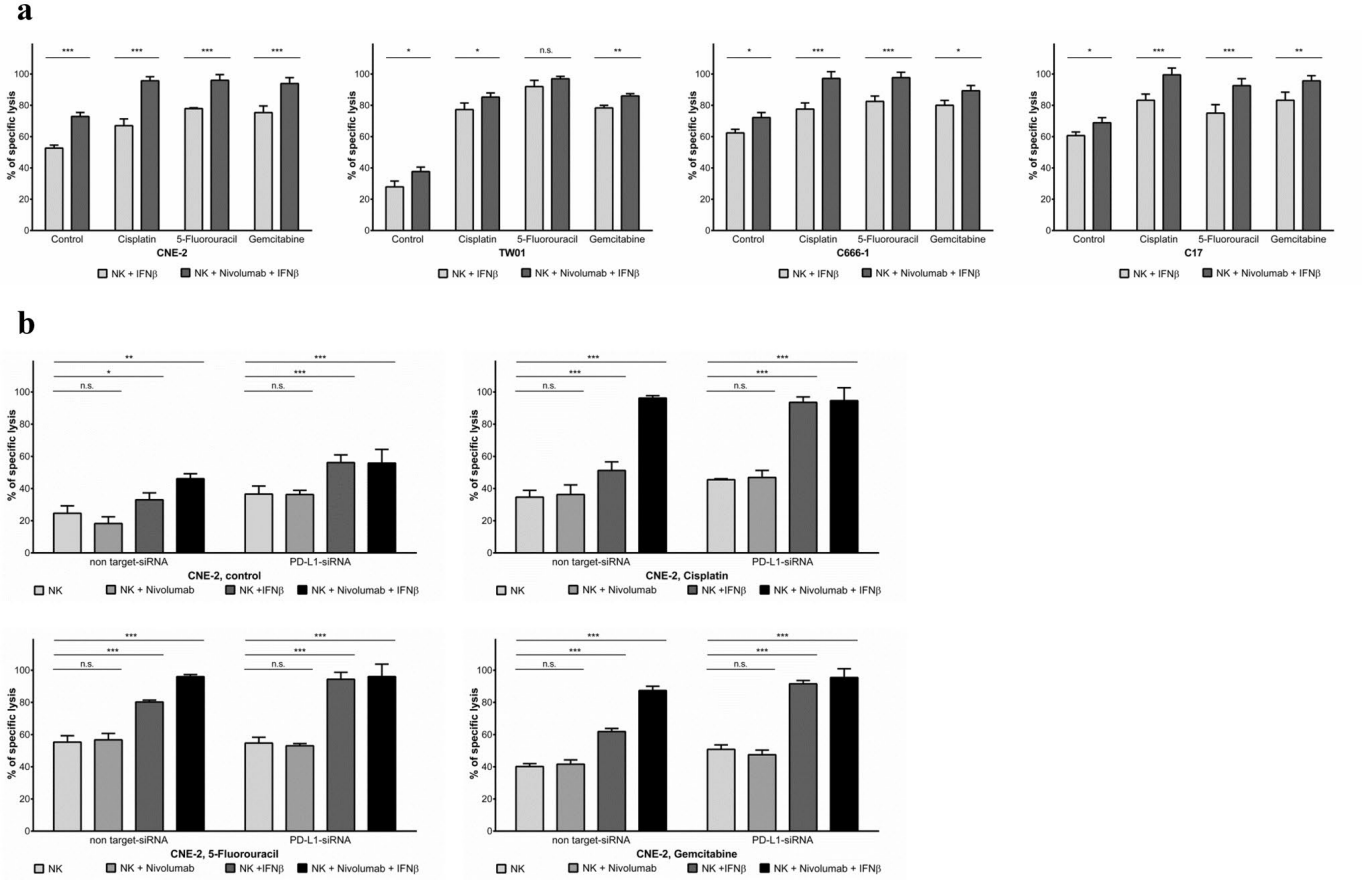
Inhibition of PD-1 increases killing of NPC cells by activated NK cells.** a** Pre-treatment of IFNβ-activated NK cells with the anti-PD-1 antibody nivolumab and subsequent exposure to NPC cells pretreated with chemotherapeutics. **b** Transfection of NPC cell line CNE-2 with PD-L1 siRNA or non-target siRNA and subsequent exposure to chemotherapeutics before co-culture with IFNβ-stimulated NK cells. Cytotoxicity was measured by calcein release assay. Data are presented as means ± S.E.M. Asterisks indicate statistically significant differences between all cell lines in one ratio-group (two-way ANOVA; *P < 0.05; **P < 0.01; ***P < 0.001)

Since chemotherapeutic agents induce PD-L1 expression on NPC cells, and IFNβ has previously been shown to induce PD-1 expression on NK cells [16], our next aim was to establish the contribution of the PD-L1/PD-1 checkpoint to the killing of NPC cells exposed to chemotherapeutics by activated NK cells. To study this, IFNβ-pre-treated NK cells were incubated with the anti-PD-1 antibody nivolumab for 1 h before co-culturing them with NPC cells pre-treated with anticancer agents. Cytotoxicity was determined by the calcein-release assay. As demonstrated in Fig. 3a, blocking of PD-1 increased the sensitivity of pre-treated NPC cells to the cytolytic activity of activated NK cells. Nivolumab only slightly increased the killing of untreated NPC cells brought about by IFNβ-pre-treated NK cells and had no significant effect on the killing of chemotherapy-pretreated NPC cells by non-activated NK cells (Suppl. Figure 4). To evaluate whether the observed increase in the killing of NPC cells by IFNβ-activated NK cells when combined with nivolumab was due to the blockade of the PD-L1/PD-1 interaction, PD-L1 expression in NPC cells was silenced by specific siRNA; the efficiency of siRNAs in silencing PD-L1 expression was monitored by flow cytometry (Suppl. Figure 5B). As shown in Fig. 3b and Suppl. Figure 5A, silencing of PD-L1 significantly increased the killing of NPC cells pretreated with cisplatin, 5-fluorouracil or gemcitabine by NK cells activated with IFNβ. No significantly higher level of NPC killing was seen in NPC cells not treated with chemotherapeutics or cells treated with non-target siRNA. Silencing of PD-L1 increased cytotoxicity to a similar degree to that achieved by the addition of nivolumab to non-target controls. These results suggest that the sensitizing effect of anticancer drugs on the killing of NPC cells by activated NK cells can be augmented by PD-L1/PD-1 checkpoint blockade.

### Inhibition of the PD-L1/PD-1 checkpoint increases susceptibility of NPC cells to cytotoxicity of NK cells treated with anticancer drugs.

**Fig. 4:**
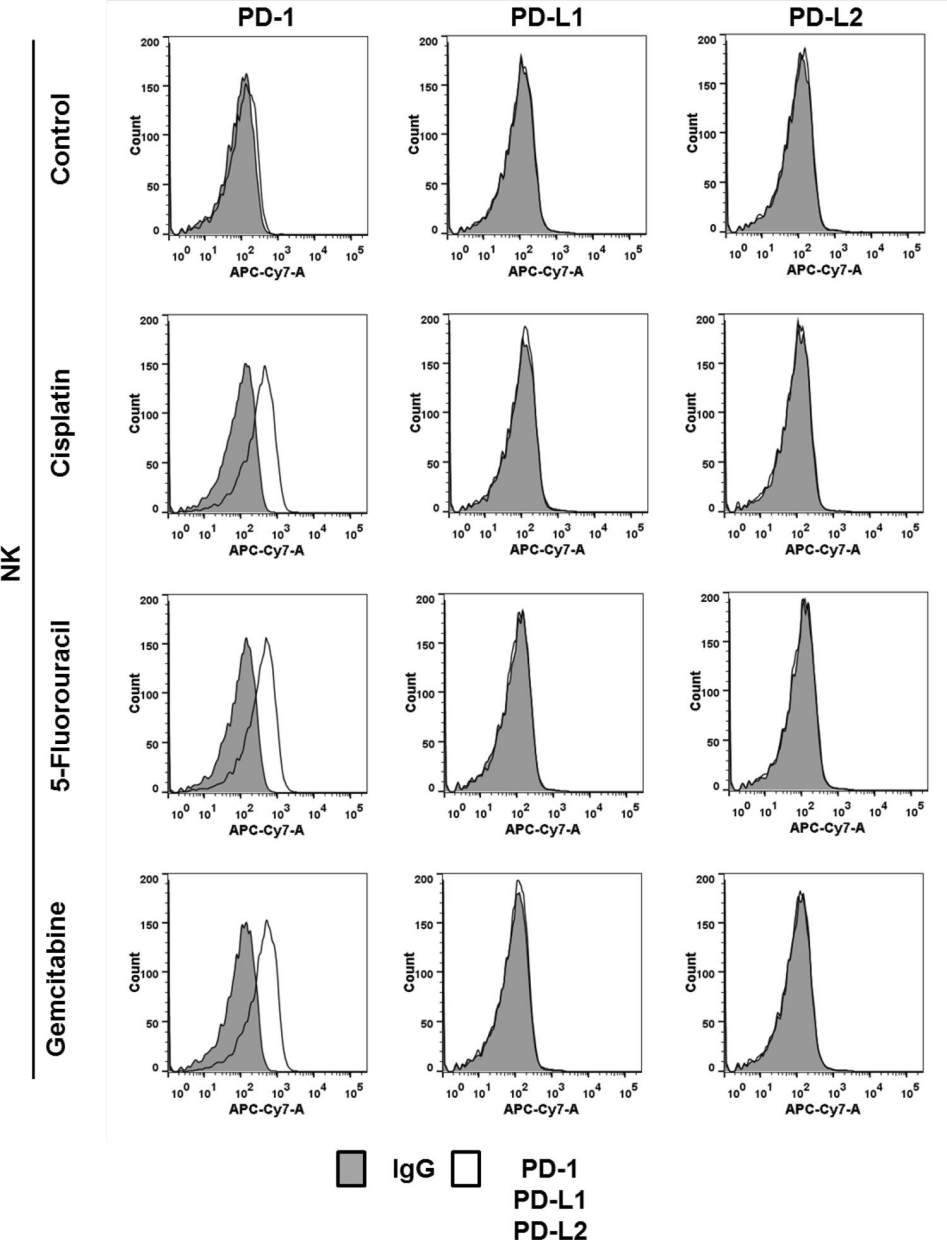
Chemotherapy upregulates surface expression of PD-1 in NK cells. Cells were incubated with cisplatin (2.5 µg/ml), 5-fluorouracil (32 µg/ml) or gemcitabine (10 µg/ml) for 24 h and PD-1, PD-L1 and PD-L2 expression was analyzed by flow cytometry

**Fig. 5:**
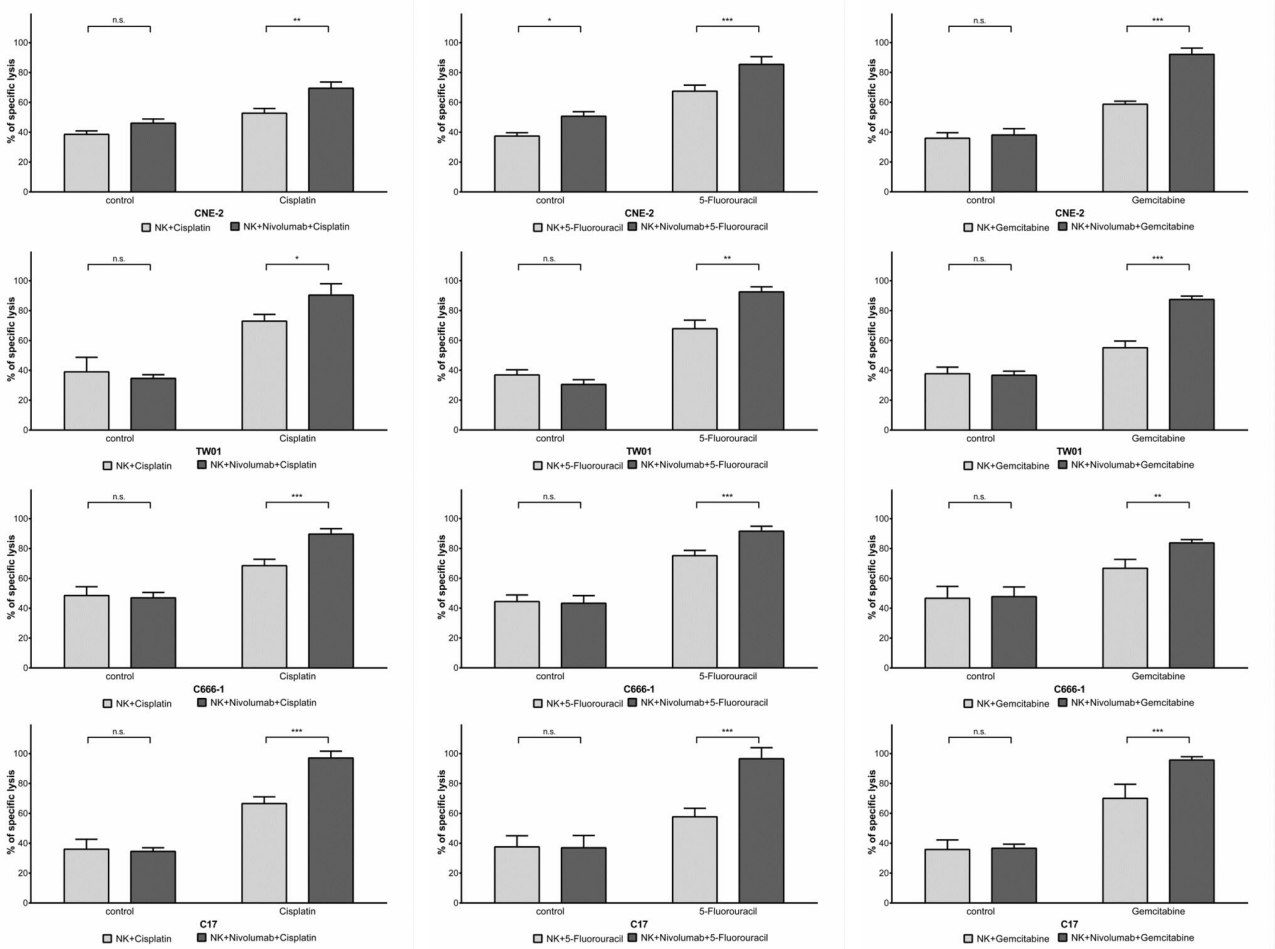
Inhibition of PD-1 increases killing of NPC cells by NK cells in the presence of chemotherapeutics. NPC and NK cells were incubated with cisplatin (2.5 µg/ml), 5-fluorouracil (32 µg/ml) or gemcitabine (10 µg/ml) for 24 h. Target cells were labeled with calcein, plated in a 96-well plate and incubated with NK cells for 4 h at an E:T ratio of 6:1. Lysis of target cells was determined by measurement of calcein in collected supernatants by an ELISA reader. Data are presented as means ± S.E.M. Asterisks indicate statistically significant differences between all cell lines in one ratio-group (two-way ANOVA; *P < 0.05; **P < 0.01; ***P < 0.001)

In patients receiving chemotherapy, all cells, including NK cells, are exposed to the effect of chemotherapeutics. The next question we wished to address was therefore whether chemotherapeutics could also influence the expression of PD-1 and PD-L1 in NK cells. NK cells were incubated for 24 h with cisplatin, 5-fluorouracil or gemcitabine and surface expression of the PD-L1/PD-1-checkpoint members was analyzed by flow cytometry. All three drugs increased expression of PD-1, but not PD-L1 and PD-L2 in NK cells (Fig. 4). Since chemotherapeutics induced PD-1 expression on NK cells and PD-L1 expression in NPC cells, we next investigated whether a blockade of the PD-L1/PD-1 checkpoint would increase the cytotoxicity of NK cells against NPC cells when both were treated with chemotherapeutics. As shown in Fig. 5, chemotherapy did not alter NK-cell cytotoxicity towards NPC cells. However, when the PD-1 blocking antibody nivolumab was added to NK cells pretreated with anticancer drugs, the cytotoxicity towards NPC cells was significantly increased. Taken together, our results show that disrupting the PD-L1/PD-1 interaction enhances the susceptibility of NPC cells to NK-cell cytotoxicity in the context of chemotherapy.

### Chemotherapy induces PD-L1 expression in NPC cells and PD-1 expression in NK cells through the NF-κB pathway

**Fig. 6:**
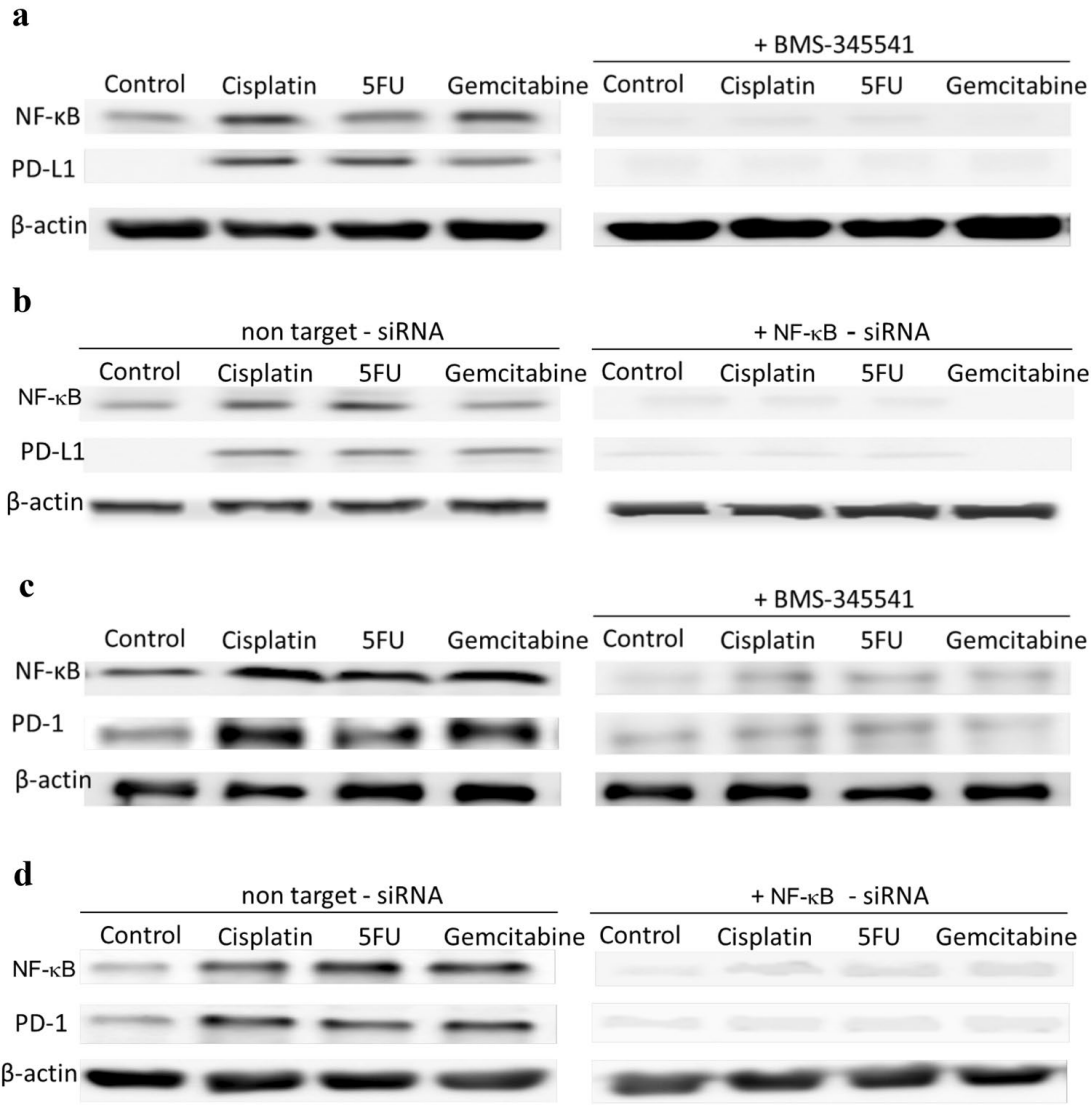
Chemotherapeutics induce PD-L1 expression in NPC cells and PD-1 expression in NK cells via upregulation of NF-κB. NPC cells **a** or NK cells **c** were incubated with the NF-κB inhibitor BMS-345541 for 1 h before incubation with chemotherapeutics. NPC cells were transfected with NF-κB siRNA **b** or NK cells with NF-κB siRNA **d** for 16 h and then incubated with chemotherapeutics. Expression of NF-κB, PD-L1 and PD-1 was analyzed by immunoblot

Chemotherapeutics have been shown to modulate gene expression through various signaling pathways, including the NF-κB pathway [17]. Incubation of ovarian cancer cells with gemcitabine or paclitaxel led to the upregulation of PD-L1 [17]. In addition, the PD-1 promoter has been found to have a NF-κB response element [25]. We therefore asked the question whether the NF-κB pathway was involved in the upregulation of PD-L1 in NPC cells and PD-1 in NK cells after incubation with anticancer agents. To study this, NPC cells and NK cells were pre-treated in the presence or absence of the NF-κB inhibitor BMS-345541 and 1 h later exposed to cisplatin, 5-fluorouracil or gemcitabine. Activation of NF-κB was seen in both NK and NPC cells when not treated with BMS-345541 after 24 h incubation with chemotherapeutic agents, as shown by immunoblot (Fig. 6a, c; Suppl. Figure  6A, C) or flow cytometry (Suppl. Figure 7A; Suppl. Figure 8A). NF-κB expression was associated with expression of PD-L1 in NPC cells (Fig. 6a; Suppl. Figure 7B) and PD-1 in NK cells (Fig. 6c; Suppl. Figure 8B). Incubation of cells with BMS-345541 completely suppressed the activation of NF-κB and expression of PD-L1 and PD-1 (Fig. 6a, c; Suppl. Figure 7A, B; Suppl. Figure 8A, B). To further investigate the role of the NF-κB pathway in this model, NPC cells and NK cells were incubated with NF-κB-specific siRNA and expression of PD-L1 in NPC cells and PD-1 in NK cells was determined by immunoblot; the efficiency of the siRNA silencing of NF-κB was monitored by flow cytometry (Suppl. Figure 7C; Suppl. Figure 8C). Silencing of NF-κB downregulated PD-L1 expression in NPC cells (Fig. 6b; Suppl. Figure 6B) and PD-1 expression in NK cells (Fig. 6d; Suppl. Figure 6D). These results suggest that anticancer agents upregulate PD-L1 and PD-1 in a cell-specific way via activation of the NF-κB pathway.

## Discussion

In this paper, we demonstrate that (1) chemotherapeutics cisplatin, 5-fluorouracil and gemcitabine sensitize NPC cells to NK-cell killing, that (2) chemotherapeutics upregulate expression of PD-L1 in NPC cells and PD-1 in NK cells via the NF-κB-pathway, and that (3) blocking of the PD-L1/PD-1 checkpoint further increases the killing of NPC cells by NK cells in the context of anticancer agents. In addition, we show that IFNβ increases the cytotoxic activity of NK cells against NPC cells treated with chemotherapeutics and that this effect can also be augmented by disrupting the PD-L1/PD-1 interaction. Our results point to a potential clinical benefit of introducing PD-L1/PD-1 checkpoint blockade into chemotherapy regimens for patients with NPC and suggest that the adoptive transfer of activated NK cells could be beneficial in this setting.

Chemotherapeutic drugs can alter the local immune state, affecting the capacity to induce a tumor‐specific response [26]. Recently, it was shown that chemotherapy upregulates immune checkpoint signals like the PD-1/PD-1 ligand 1 axis in certain tumors [27]. Upregulation of PD-L1 on tumor cells by chemotherapy may therefore allow tumors to escape from immune surveillance [28], in which case, anti-PD-L1/PD-1 checkpoint-inhibition could have a role to play in chemotherapeutic regimens. Such a benefit was shown for the first time in a randomized study in patients with non-small cell lung cancer (NSCLC), which demonstrated a significant increase in tumor response when an anti-PD-1 antibody was added to induction chemotherapy with cisplatin and permetrexate [18]. In two subsequent large randomized trials the addition of pembrolizumab, an anti-PD-1 antibody, to the chemotherapeutics significantly increased progression- and overall survival in NSCLC patients [29,30].

NK cells play a major role in tumor surveillance [24]. NPC tumors are characterized by lymphomonocytic infiltrates and the number of NK cells in tumors appears to be of prognostic significance [15]. Previously, we have shown that IFNβ, which is used as maintenance therapy in the treatment of children and adolescents with NPC, increases the ability of patients’ NK cells to kill NPC cells in vitro [16]. Here, we demonstrate that the chemotherapeutics cisplatin, 5-fluorouracil and gemcitabine, which are commonly used in the treatment of NPC patients, upregulate PD-1 on NK cells and PD-L1 on NPC cells and could therefore suppress NK-cell killing.

PD-1 is mainly expressed by activated NK and T cells. The cis-regulatory elements of the PD1 gene contain various transcription factor binding sites, including an IFN-stimulated response element (ISRE), as well as binding sites for AP-1, NFATc1, FoxO1 and NF-κB [25]. After T cell receptor activation, PD-1 expression in T-cells is predominately mediated via NFATc1. A role for NF-κB in inducing PD-1 expression has so far only been described in macrophages in response to TLR stimulation [31]. Increased expression of PD-1 on NK cells has been observed in tumor infiltrates of patients with digestive cancers and found to be associated with a poor prognosis [32].

Chemotherapeutics have been shown to induce NF-κB signaling in various cancers including nasopharyngeal cancer cells in a dose-dependent manner in vitro and in vivo [33]. The PD-L1 gene has a transcription factor binding site for NF-κB and upregulation of PD-L1 expression via NF-κB has been shown in ovarian cancer cells [17].

Our observation that pretreatment of NPC cells with chemotherapeutics increased their killing by NK cells is not surprising and has been described before in other tumor cells [24]. Chemotherapeutics sensitize tumor cells to NK cells by various mechanisms such as modulating the expression of NK-cell activating ligands, upregulation of death receptors or production of damage-associated molecule patterns (DAMP) [24].

In our experiments, incubation of NK cells from healthy donors with chemotherapeutics cisplatin, 5-fluorouracil or gemcitabine did not alter the degree of killing of NPC cells. Data on NK-cell function during chemotherapy are conflicting and depends in part on the chemotherapeutics applied [34]. In women with breast cancer, NK-cell killing following neoadjuvant chemotherapy containing 5-fluorouracil, epirubicin, cyclophosphamide or docetaxel was increased in 24 out of 39 patients and decreased in 15 of them [35].

Although NK-cell activity in humans varies depending on factors like age, weight, smoking, alcohol consumption and diet [36,37], we did not see major differences in our main results using NK cells from different donors. Taken together, the data suggest that NK-cell function is preserved in patients undergoing chemotherapy, that NPC tumors cells exposed to chemotherapeutics show increased sensitivity to NK-cell killing and that the degree of this killing can be augmented by the addition of an anti-PD-1 antibody during chemotherapy. The use of anti-PD-1 therapy in patients with NPC is further supported by the results of two phase I/II studies with anti-PD-1 antibody alone in patients with refractory NPC resulting in overall response rates of 20.5–25.9% [9,10]. In addition, high expression of PD-1 and co-expression of PD-L1 in NPC tumors has been shown to be associated with poor outcome [38].

Several clinical trials involving the adoptive transfer of either autologous or allogeneic NK cells have been performed in patients with cancer in recent years [39]. Infusion of large doses of NK cells has generally been shown to be safe and efficacious [40,41]. Based on our results, a preferable approach would be the transfer of ex vivo-activated NK cells together with an anti-PD-L1/PD-1 checkpoint inhibitor to patients undergoing chemotherapy. Such an approach is feasible, as demonstrated by the combination of radiochemotherapy with NK-cell transfer followed by PD-1 inhibition in a patient with NSCLC, which led to long-term tumor control [42]. Adoptive transfer of NK cells in combination with chemotherapy has also been turned out to be safe and to improve outcome in patients with locally advanced colon carcinoma [43].

In this study, the highest degree of killing was observed when chemotherapy-pretreated NPC cells were exposed to IFNβ-activated NK cells in the presence of anti-PD-1 antibody. Chemotherapeutics such as anthracyclines, bortezomib, oxaliplatin, but not cisplatin have been shown to elicit immunogenic cell death via induction of type I interferon signaling in tumor cells [34]. In several independent cohorts of patients with breast cancer, a type I interferon-related signature correlated with response to anthracycline-based chemotherapy [46]. Addition of exogenous type I interferon to cisplatin in a mouse melanoma model induced a protective immune response similar to the one by anthracyclines alone [46]. As cisplatin is a major component of chemotherapy regimens in NPC, and protocols using IFNβ as maintenance therapy have an inferior rate of systemic relapses [7], further studies analyzing the effect of IFNβ on the activity of chemotherapeutics in NPC should be of major interest.

In conclusion, we have shown that chemotherapeutics used for the treatment of NPC sensitize NPC cells to killing by NK cells and that this lethal action can be augmented by blockade of the PD-L1/PD-1 checkpoint, which in turn is induced by chemotherapeutics through NF-κB-dependent upregulation of PD-1 in NK cells and PD-L1 in NPC cells. Thus, the integration of an anti-PD-1 antibody into chemotherapeutic regimens for NPC patients and into protocols with adoptive NK-cell transfer may offer a novel and effective means of improving treatment strategies in NPC.

## Electronic supplementary material

Below is the link to the electronic supplementary material.

Supplementary file1 (PDF 3709 kb)

## Abbreviations

Calcein-AM: Calcein-acetyoxymethyl
EBV: Epstein–Barr virus
GPOH: German Society of Pediatric Oncology and Hematology
IFNβ: Interferon beta
NF-κB: Nuclear factor kappa B
NPC: Nasopharyngeal carcinoma
NSCLC: Non-small cell lung cancer
PD-1: Programmed cell death protein 1
PD-L1: Programmed cell death ligand 1
PdX: Patient-derived xenograft
RFU: Relative fluorescent units
