# Supplementary material for: Anti-PD-1 antibody increases NK cell cytotoxicity towards nasopharyngeal carcinoma cells in the context of chemotherapy-induced upregulation of PD-1 and PD-L1
Source: Cancer Immunol Immunother. 2020 Jul 31;70(2):323–36. doi: 10.1007/s00262-020-02681-x (PMC7889576; doi:10.1007/s00262-020-02681-x)
Supplement: Supplementary file 1 — Supplementary file1 (PDF 3709 kb) [file 262_2020_2681_MOESM1_ESM.pdf]

Supplementary Figure 1

Effect of chemotherapy on cell viability and cell apoptosis. a NPC cells of cell lines were incubated with different concentrations of the indicated chemotherapeutics. Cell viability was determined by MTT test between 24 h and 72 h after begin of exposure. PdX cells C17 were only tested at one concentration per drug and compared to CNE-2 cells. Data are presented as means ± S.E.M. The one-way repeated measures ANOVA documented significant changes in the percentage of living cells starting after 24 h incubation with chemotherapeutic agents (ANOVA: * = P<0.05; ** = P<0.01; *** = P<0.001). **b** NPC cells were treated as above and apoptosis was determined by flow cytometric analysis of subdiploid DNA-content between 24 h and 72 h after begin of exposure. Data are presented as means ± S.E.M., each experiment was done three times (Student's t- test: * = P<0.05; ** = P<0.01; *** = P<0.001). **c** NPC cells labeled with calcein were treated with chemotherapeutics for 24 h. Lysis of target cells was determined by measurement of calcein in collected supernatants by an ELISA reader. Data are presented as means ± S.E.M. Asterisks indicate statistically significant differences between all cell lines in one ratio-group (one-way ANOVA; *P < 0.05; **P < 0.01; ***P < 0.001).

a

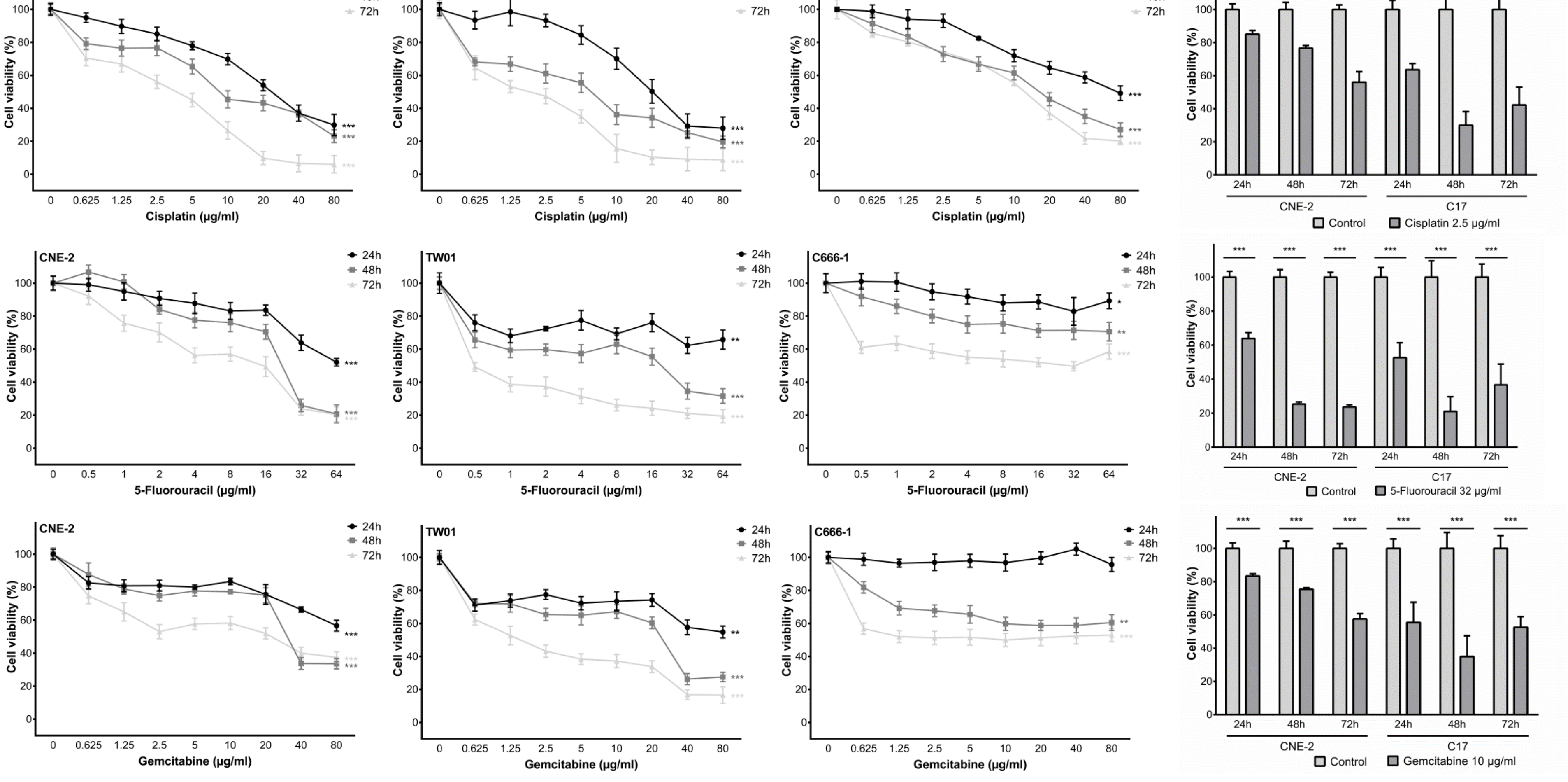

b

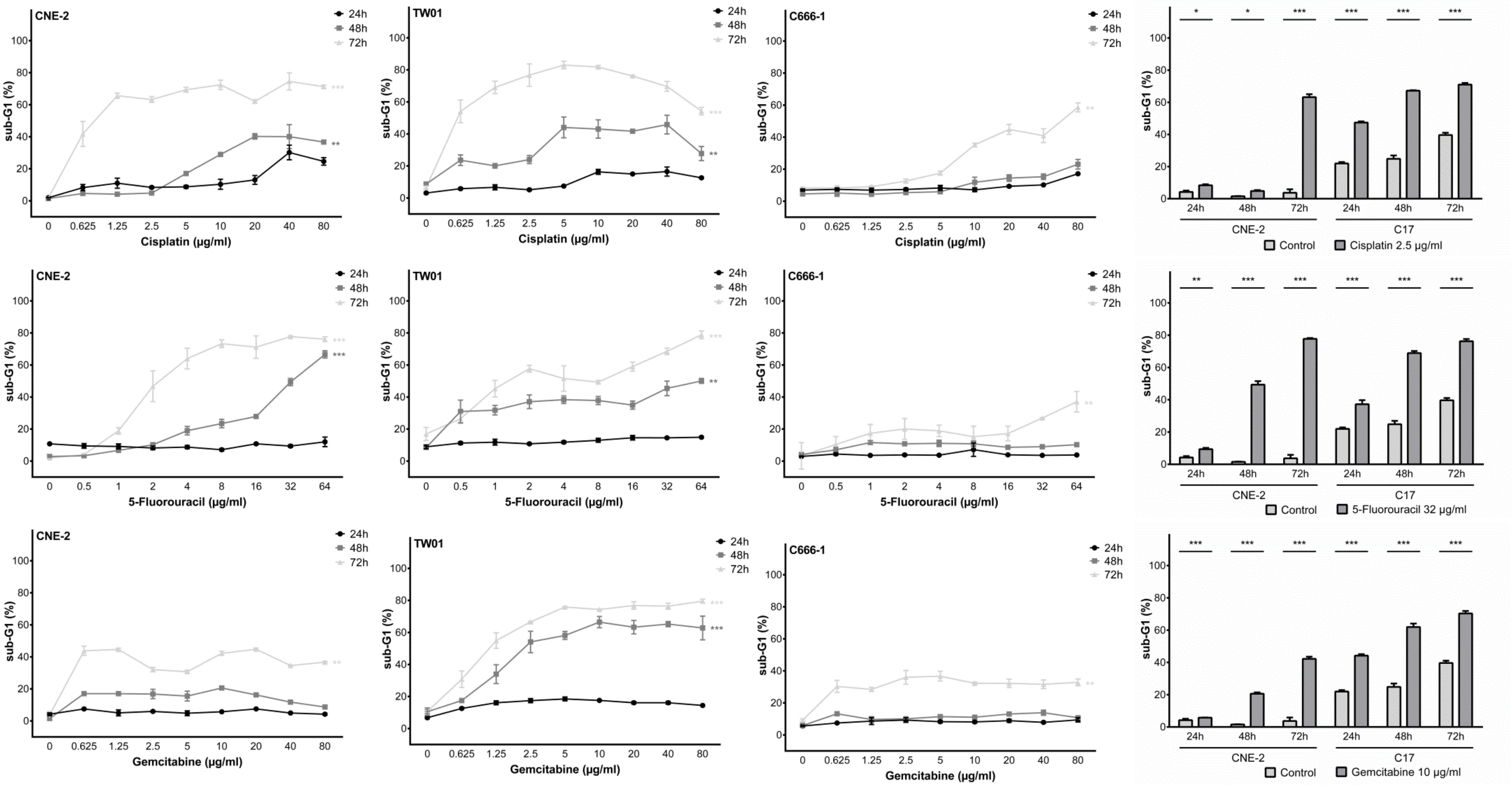
CNE-2
TW01
C666-1
24h
48h
72h
sub-G1 (%)
0
20
40
60
80
100
0 0.625 1.25 2.5 5 10 20 40 80
Cisplatin (µg/ml)
0 0.5 1 2 4 8 16 32 64
5-Fluorouracil (µg/ml)
Gemcitabine (µg/ml)
*
**
***
24h 48h 72h 24h 48h 72h
CNE-2
C17
Control
Cisplatin 2.5 µg/ml
5-Fluorouracil 32 µg/ml
Gemcitabine 10 µg/ml

c

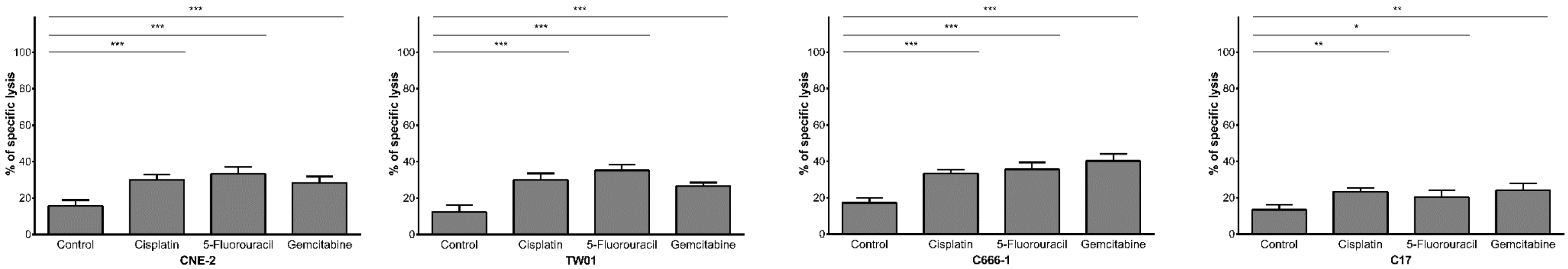

% of specific lysis
100
80
60
40
20
0
***
***
***
Control
Cisplatin
5-Fluorouracil
Gemcitabine
CNE-2
TW01
C666-1
**
*
**
C17

Supplementary Figure 2

Spontaneous lysis of NPC cell lines. NPC cells were pretreated with chemotherapeutics for 24 h and then labeled with calcein. Spontaneous lysis of NPC cells was determined by measurement of calcein in collected supernatants by an ELISA reader. The lysis was measured 4 h after cells were labeled with calcein. Data are presented as means ± S.E.M. Asterisks indicate statistically significant differences between all cell lines in one ratio-group (one way ANOVA; *P < 0.05; **P < 0.01; ***P < 0.001).

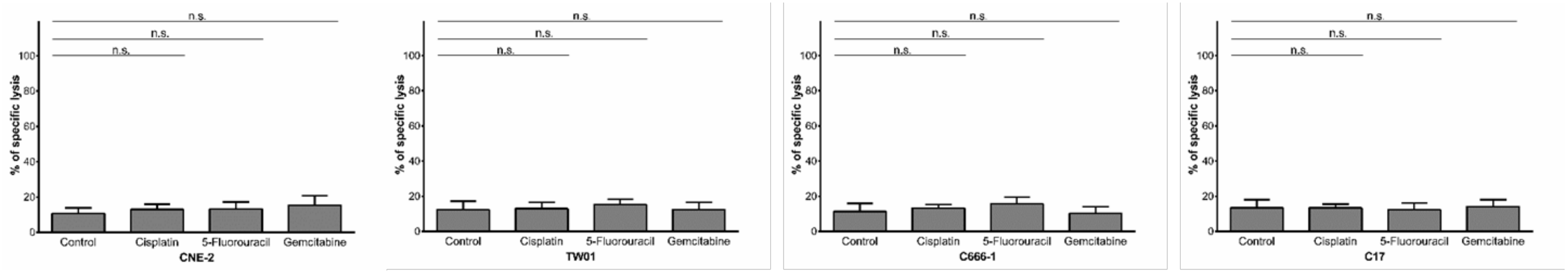

Supplementary Figure 3

Effect of IFNβ on NK cell viability and cell apoptosis. NK cells were incubated with 1,000 U/ml IFNβ. Apoptosis was determined by flow cytometric analysis of subdiploid DNA-content after 24 h from begin of exposure. Data are presented as single specimen **(a)** and as means ± S.E.M. **(b);** each experiment was done three times (Student's t- test: * = P<0.05; ** = P<0.01; *** = P<0.001). **c** NK cells were treated as above and cell viability was determined by MTT test after 24 h from begin of exposure. Data are presented as means ± S.E.M. The one-way repeated measures ANOVA documented significant changes in the percentage of living cells after 24 h incubation with IFNβ (ANOVA: * = P<0.05; ** = P<0.01; *** = P<0.001).

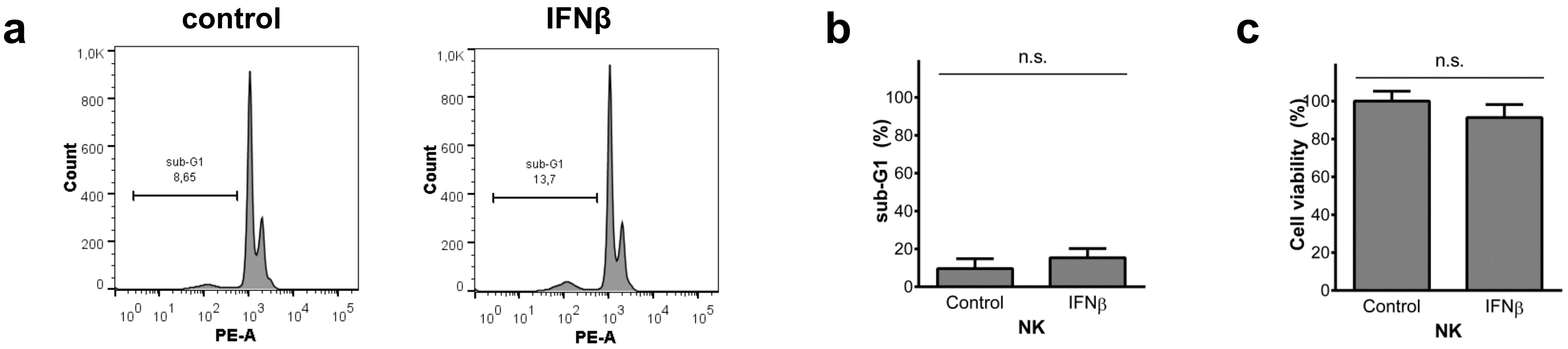

Supplementary Figure 4

No influence of PD-1 inhibition on the cytotoxicity of non-activated NK cells against NPC cells treated with chemotherapeutic agents. NK cells were treated or not with the anti-PD-1 antibody nivolumab (10 µg/ml) for 1 h and then co-cultured for 4 h with NPC cells pretreated with chemotherapeutics and labeled with calcein. Lysis of target cells was determined by measurement of calcein in collected supernatants by an ELISA reader. Data are presented as means ± S.E.M. Asterisks indicate statistically significant differences between all cell lines in one ratio-group (two-way ANOVA; *P < 0.05; **P < 0.01; ***P < 0.001).

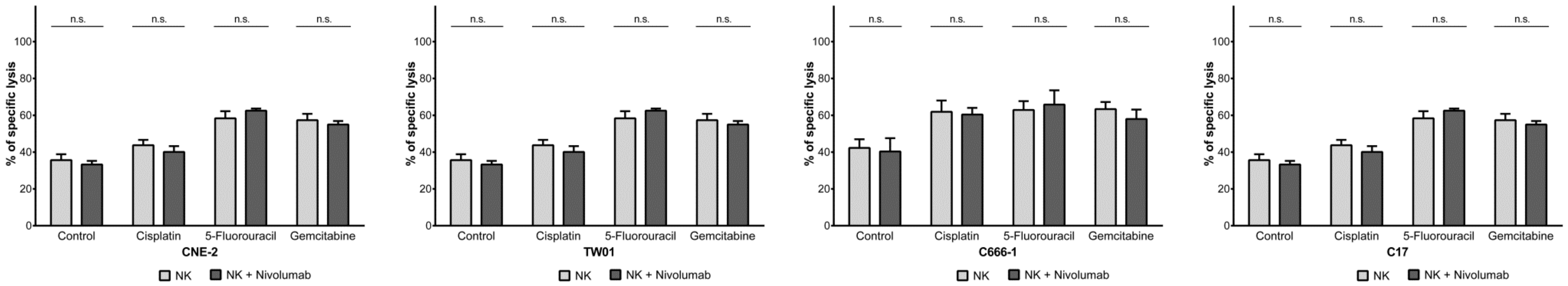

Supplementary Figure 5

Transfection of NPC cells with PD-L1 siRNA. a Transfection of NPC cells C666-1, TW01 and C17 with PD-L1 siRNA or non-target siRNA and subsequent exposure to chemotherapeutics before co-culture with IFNβ-stimulated NK cells. Cytotoxicity was measured by calcein relase assay. Data are presented as means ± S.E.M. Asterisks indicate statistically significant differences between all cell lines in one ratio-group (two-way ANOVA; *P < 0.05; **P < 0.01; ***P < 0.001). **b** Cells were treated as above and PD-L1 expression of NPC cells treated with PD-L1 siRNA or non-target siRNA was analyzed by flow cytometry.

a

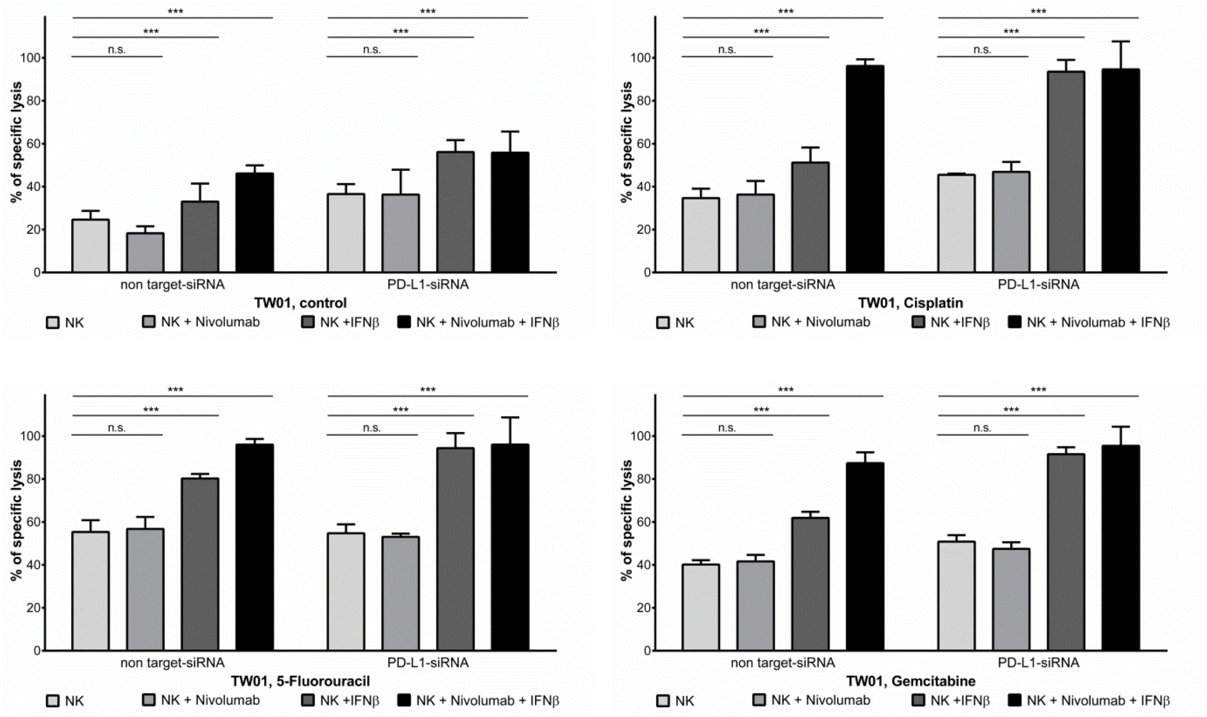

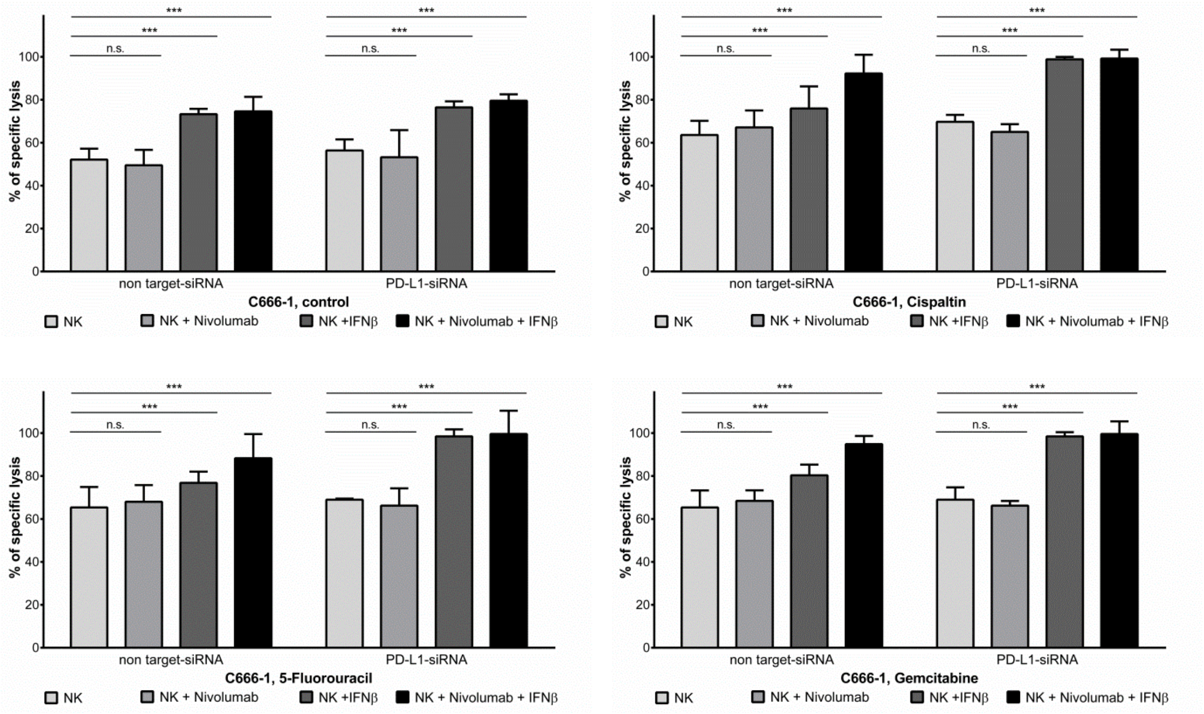

***
***
n.s.
100
80
60
40
20
0
% of specific lysis
non target-siRNA
PD-L1-siRNA
C666-1, control
C666-1, Cispaltin
C666-1, 5-Fluorouracil
C666-1, Gemcitabine
NK
NK + Nivolumab
NK +IFNβ
NK + Nivolumab + IFNβ

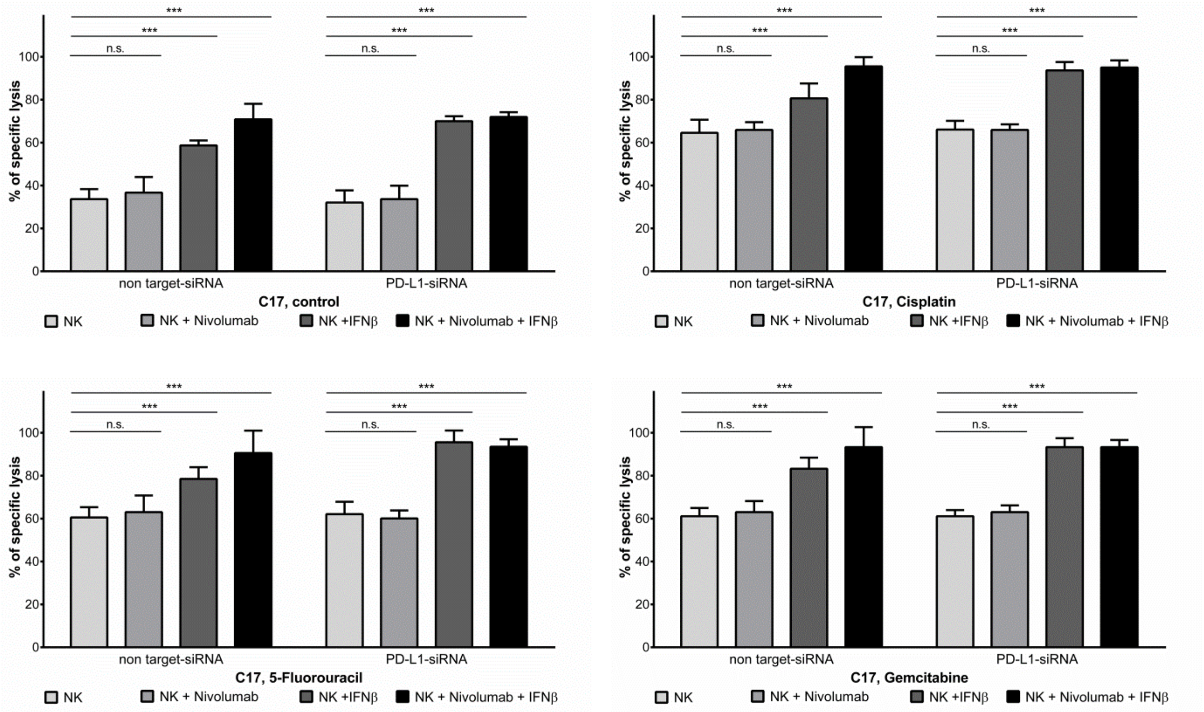

C17, control
C17, Cisplatin
C17, 5-Fluorouracil
C17, Gemcitabine
% of specific lysis
non target-siRNA
PD-L1-siRNA
n.s.
***
NK
NK + Nivolumab
NK +IFNβ
NK + Nivolumab + IFNβ

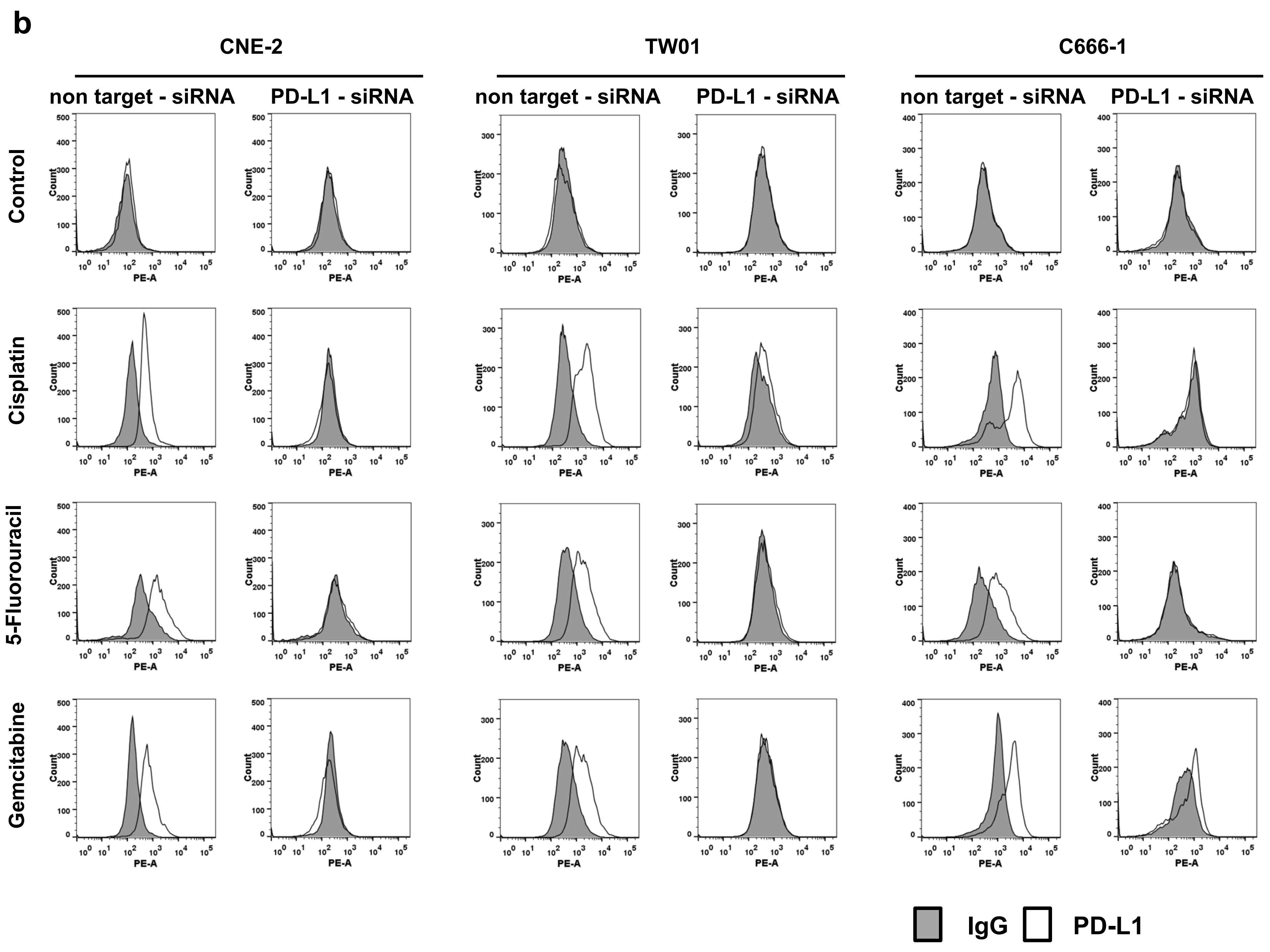

b
CNE-2
TW01
C666-1
non target - siRNA
PD-L1 - siRNA
Control
Cisplatin
5-Fluorouracil
Gemcitabine
Count
PE-A
IgG
PD-L1

Supplementary Figure 6

Relative quantification of the Western Blot from Figure 6. NPC cells **a** or NK cells **c** were incubated with the NF-κB inhibitor BMS-345541 for 1 h before chemotherapy. NPC cells **b** or NK cells **d** were transfected with NF-κB siRNA for 16 h and then incubated with chemotherapeutics.

.

a

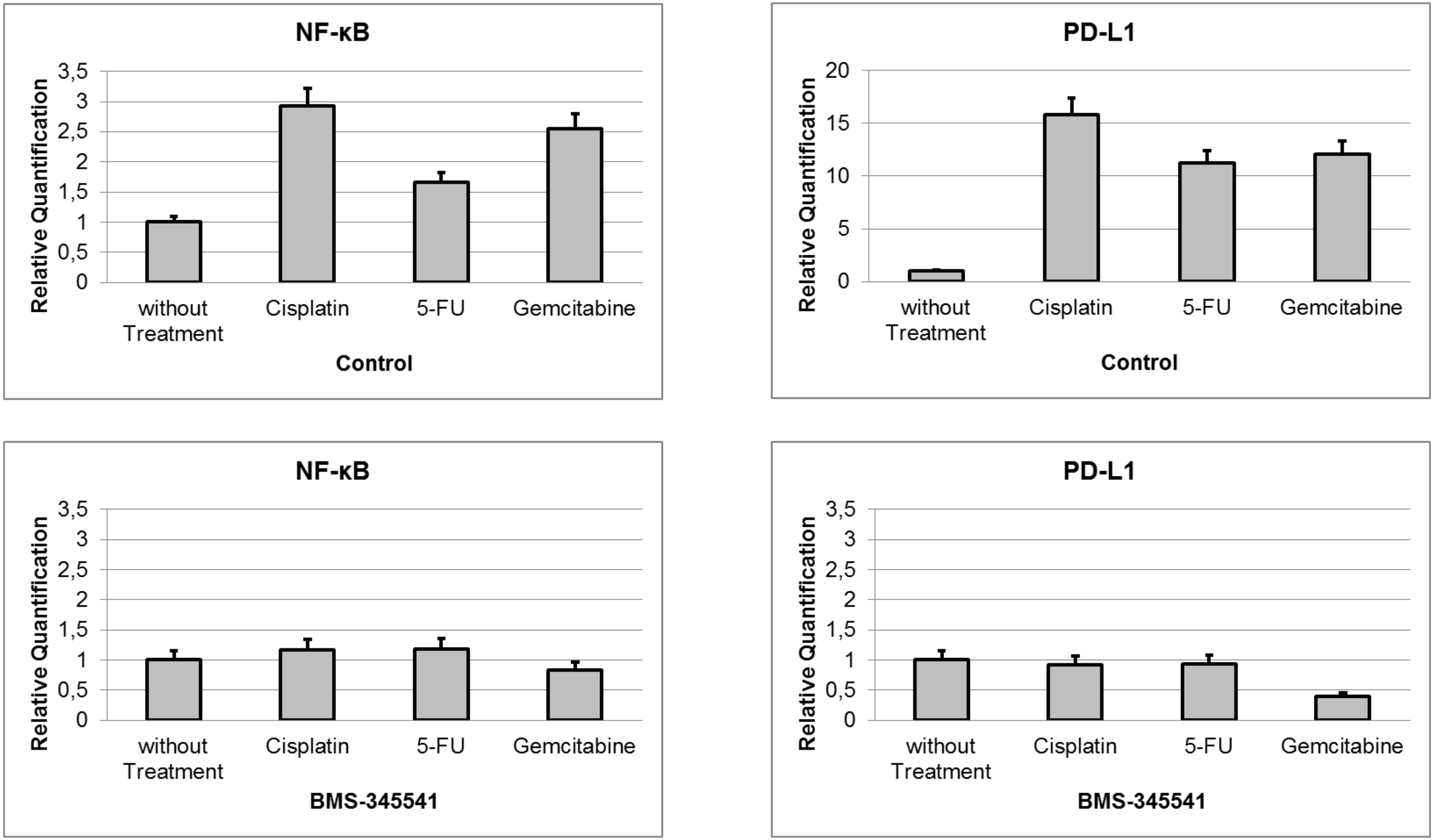

b

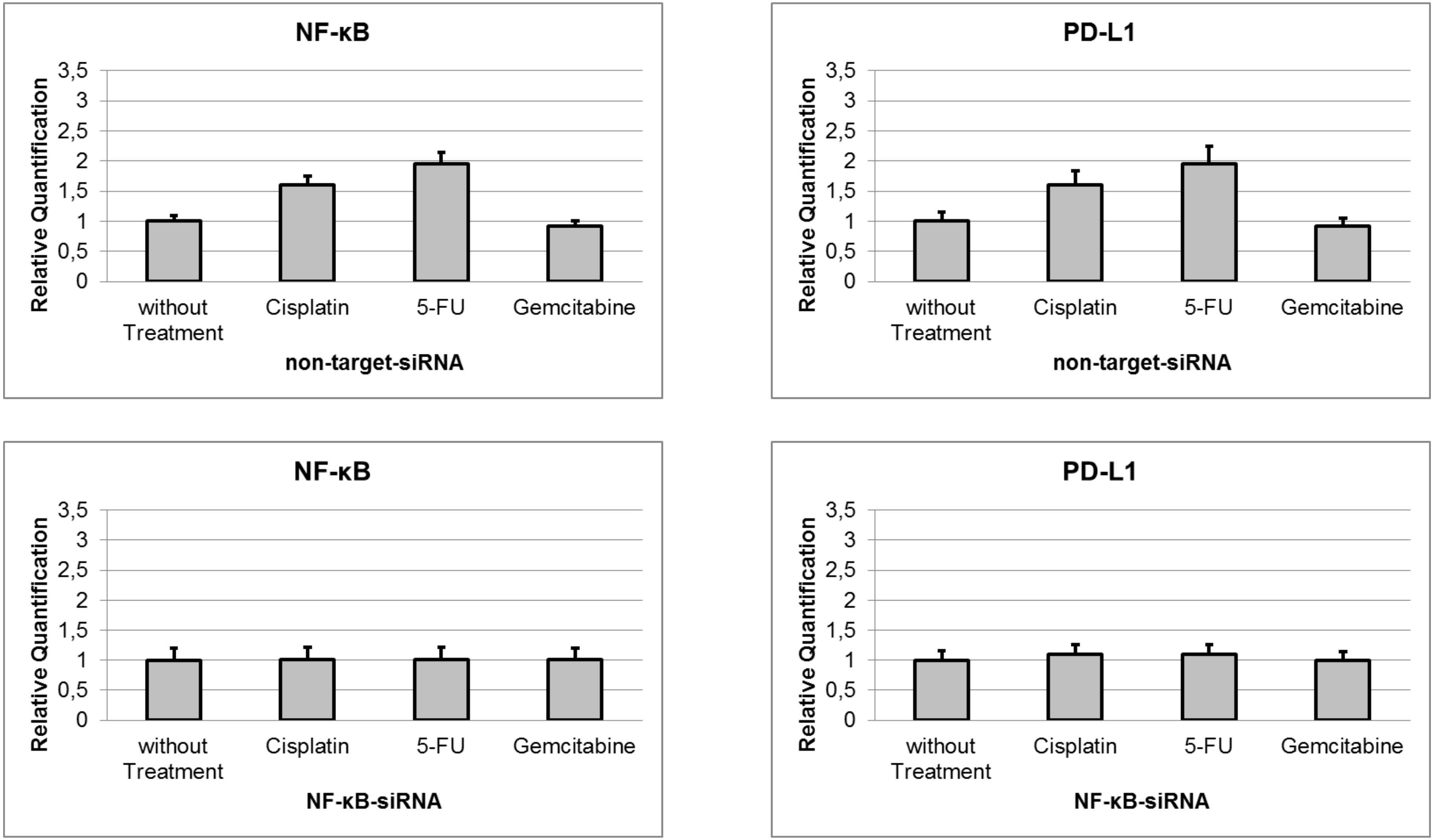
NF-κB
PD-L1
Relative Quantification
3,5
3
2,5
2
1,5
1
0,5
0
without Treatment
Cisplatin
5-FU
Gemcitabine
non-target-siRNA
NF-κB
PD-L1
NF-κB-siRNA

c

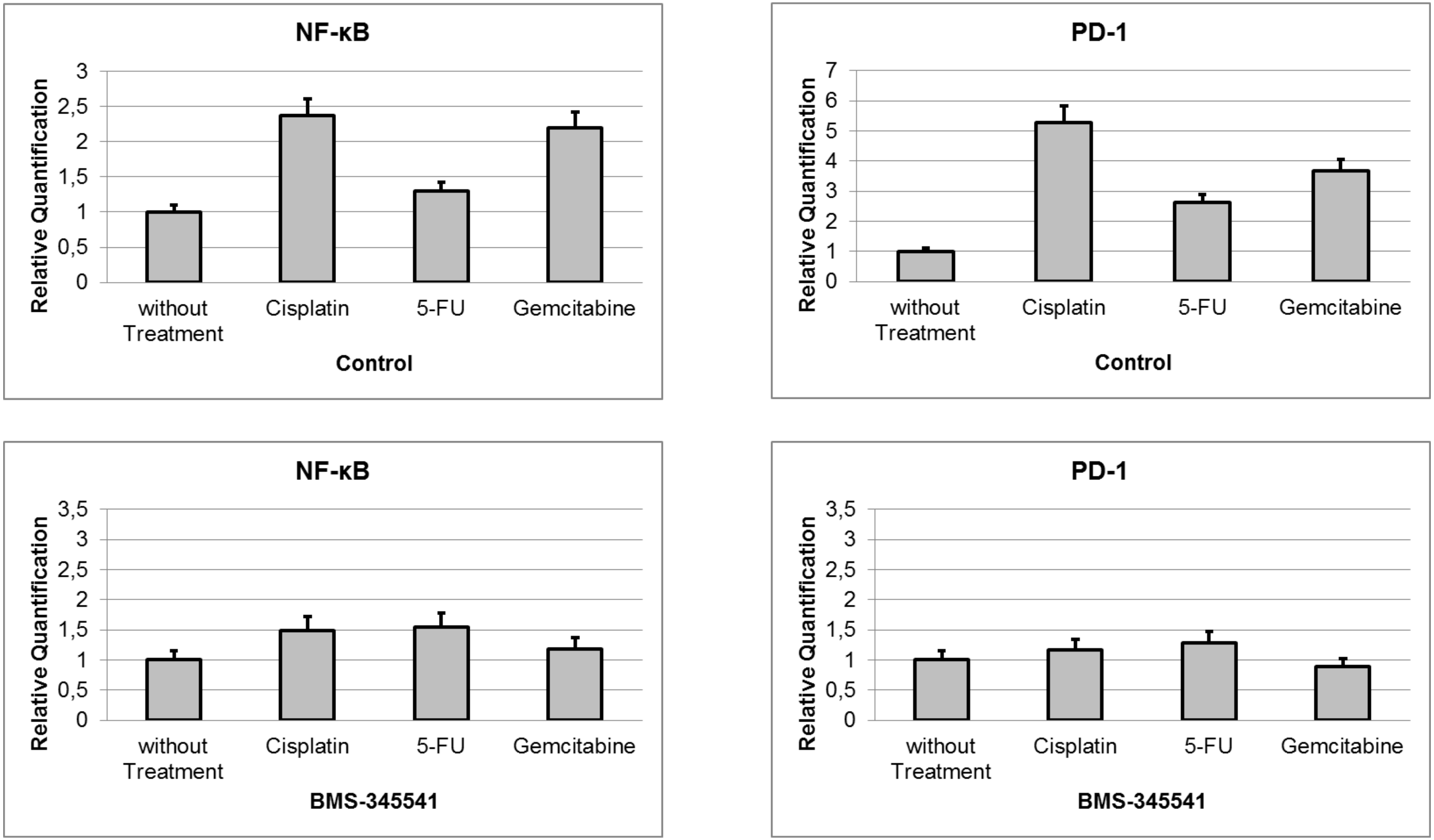
NF-κB
Relative Quantification
3
2,5
2
1,5
1
0,5
0
without Treatment
Cisplatin
5-FU
Gemcitabine
Control
PD-1
Relative Quantification
7
6
5
4
3
2
1
0
without Treatment
Cisplatin
5-FU
Gemcitabine
Control
NF-κB
Relative Quantification
3,5
3
2,5
2
1,5
1
0,5
0
without Treatment
Cisplatin
5-FU
Gemcitabine
BMS-345541
PD-1
Relative Quantification
3,5
3
2,5
2
1,5
1
0,5
0
without Treatment
Cisplatin
5-FU
Gemcitabine
BMS-345541

d

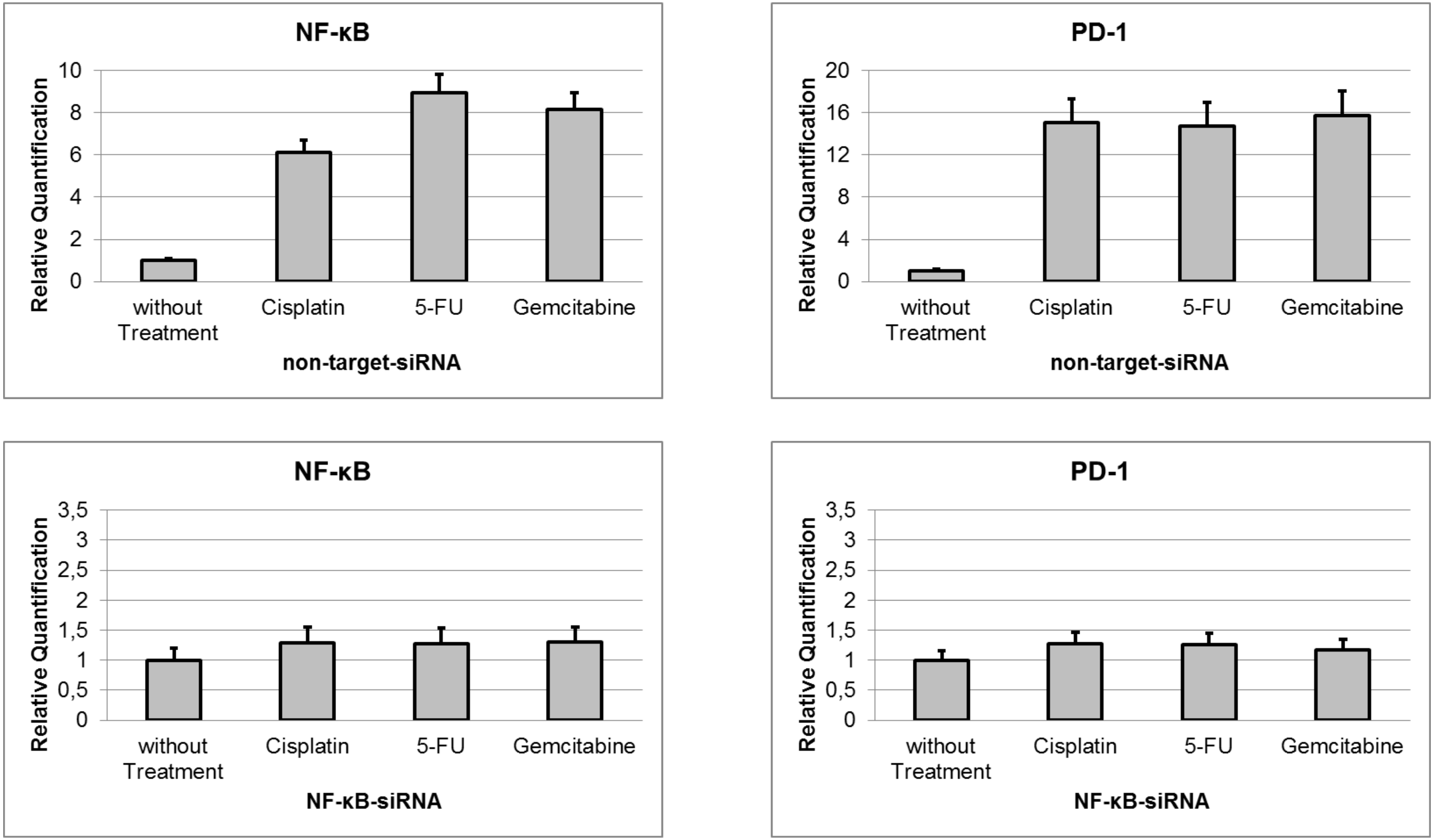
NF-κB
Relative Quantification
10
8
6
4
2
0
without Treatment
Cisplatin
5-FU
Gemcitabine
non-target-siRNA
PD-1
Relative Quantification
20
16
12
8
4
0
without Treatment
Cisplatin
5-FU
Gemcitabine
non-target-siRNA
NF-κB
Relative Quantification
3,5
3
2,5
2
1,5
1
0,5
0
without Treatment
Cisplatin
5-FU
Gemcitabine
NF-κB-siRNA
PD-1
Relative Quantification
3,5
3
2,5
2
1,5
1
0,5
0
without Treatment
Cisplatin
5-FU
Gemcitabine
NF-κB-siRNA

Supplementary Figure 7

Inhibition of NF-κB and PD-L1 expression in NPC cells treated with chemotherapeutics by NF-κB inhibitor BMS-345541 or NF-κB siRNA. NPC cells were treated with chemotherapeutics and the NF-κB inhibitor BMS-345541 for 24 h **a, b** or NF-κB siRNA or non-target siRNA **c, d**. Expression of intracellular NF-κB **a, c** or surface PD-L1 **b, d** was analyzed by flow cytometry.

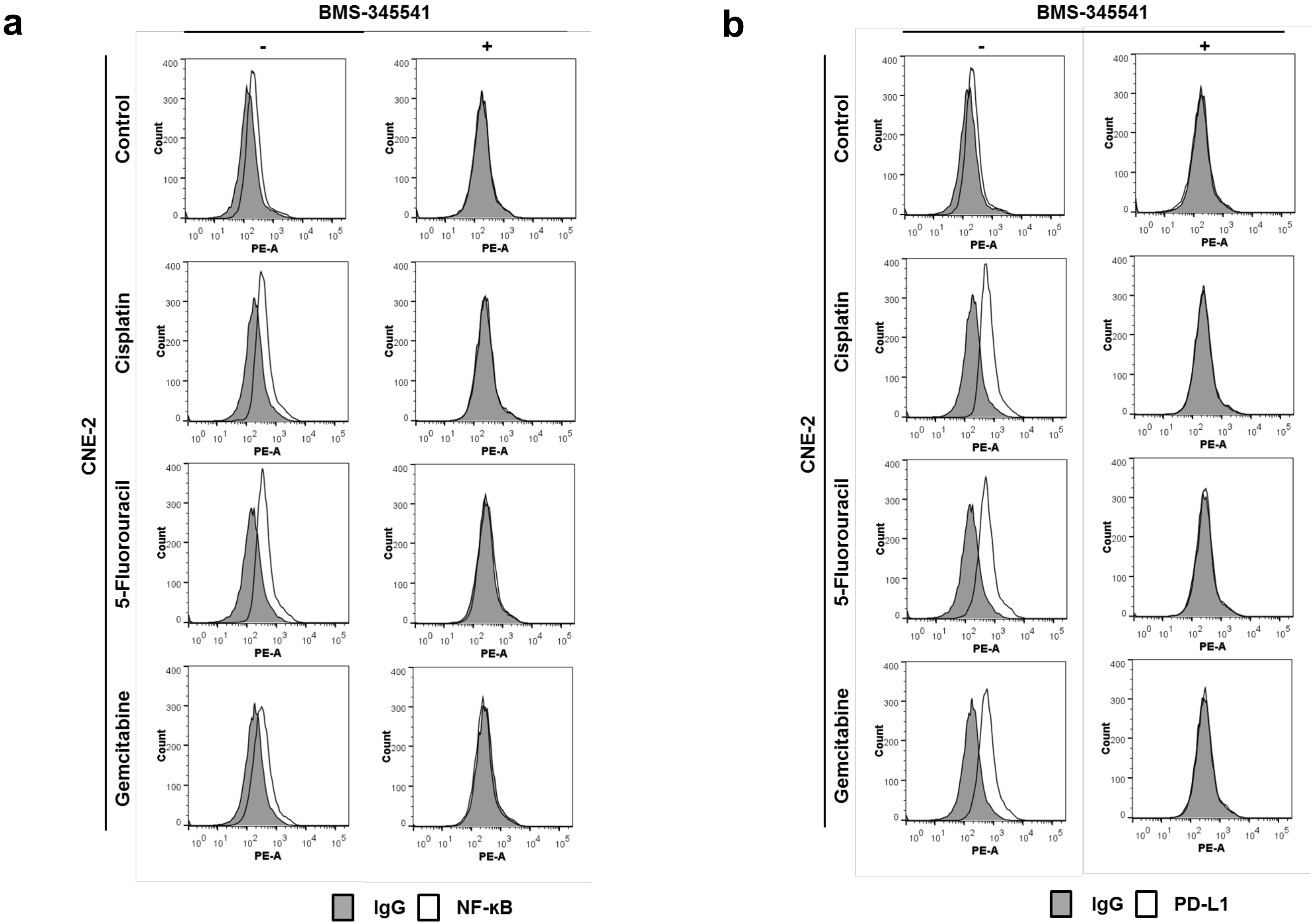

c

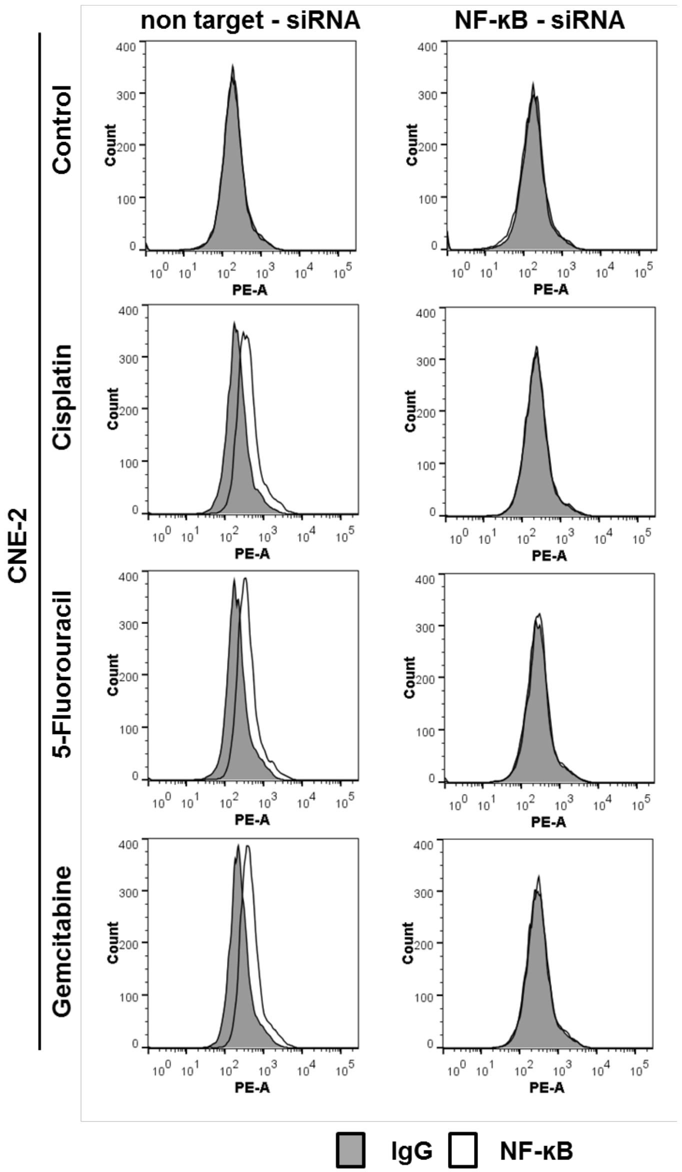

non target - siRNA
NF-κB - siRNA
CNE-2
Control
Cisplatin
5-Fluorouracil
Gemcitabine
Count
PE-A
IgG
NF-κB

d

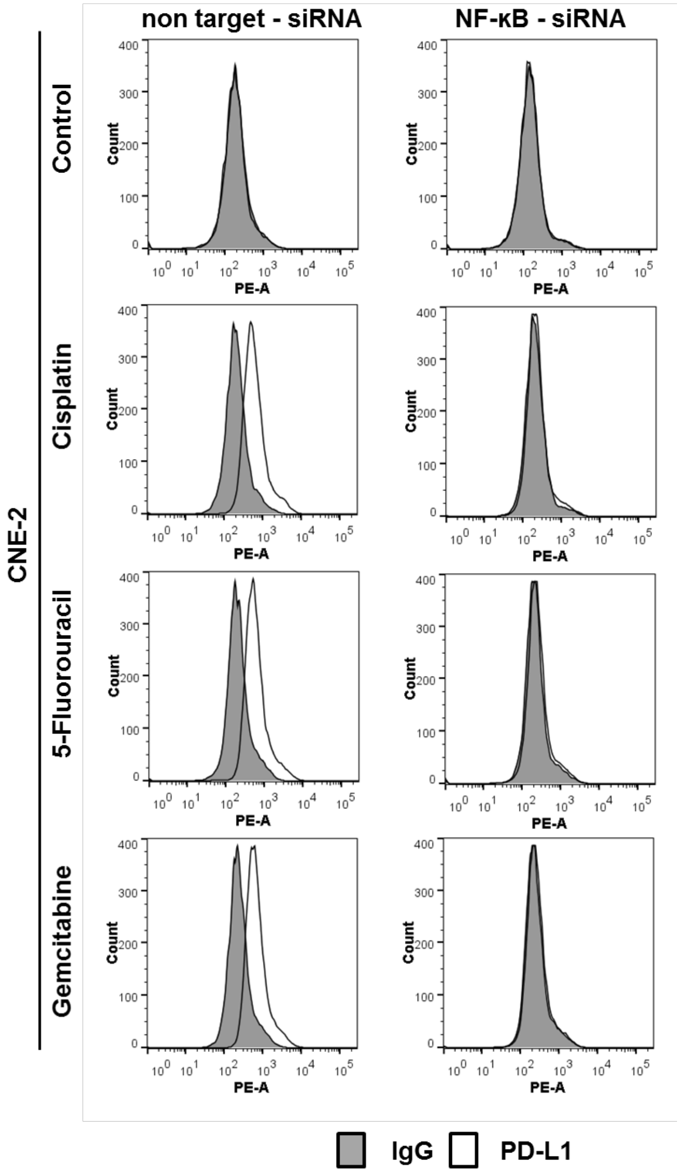

non target - siRNA
NF-κB - siRNA
CNE-2
Control
Cisplatin
5-Fluorouracil
Gemcitabine
Count
PE-A
IgG
PD-L1

Supplementary Figure 8

Inhibition of NF-κB and PD-1 expression in NK cells treated with chemotherapeutics by NF-κB inhibitor BMS-345541 or NF-κB siRNA. NK cells were treated with chemotherapeutics and the NF-κB inhibitor BMS-345541 **a, b** or NF-κB siRNA or non-target siRNA **c, d** . Expression of intracellular NF-κB **a, c** or surface PD-L1 **b, d** was analyzed by flow cytometry.

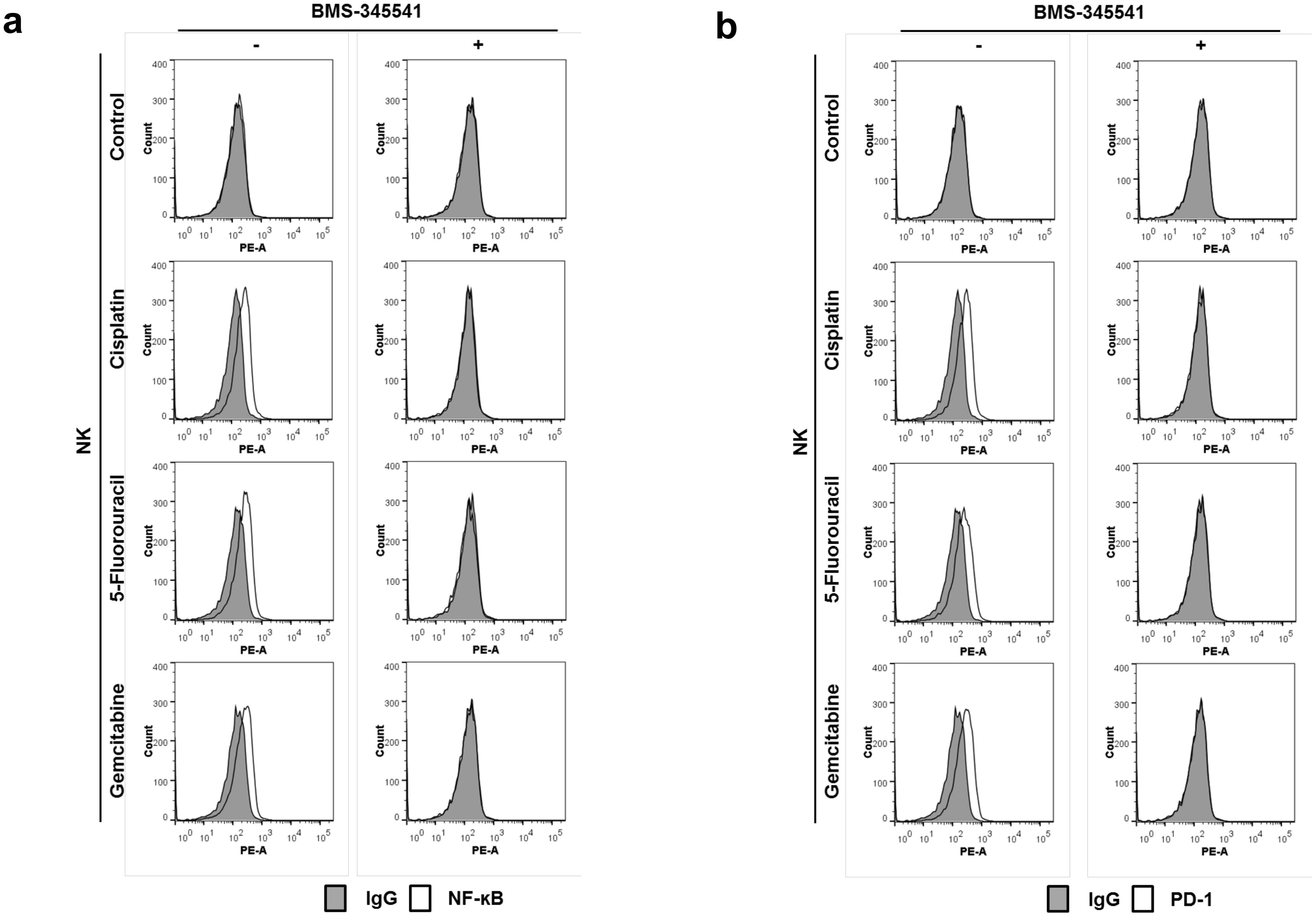

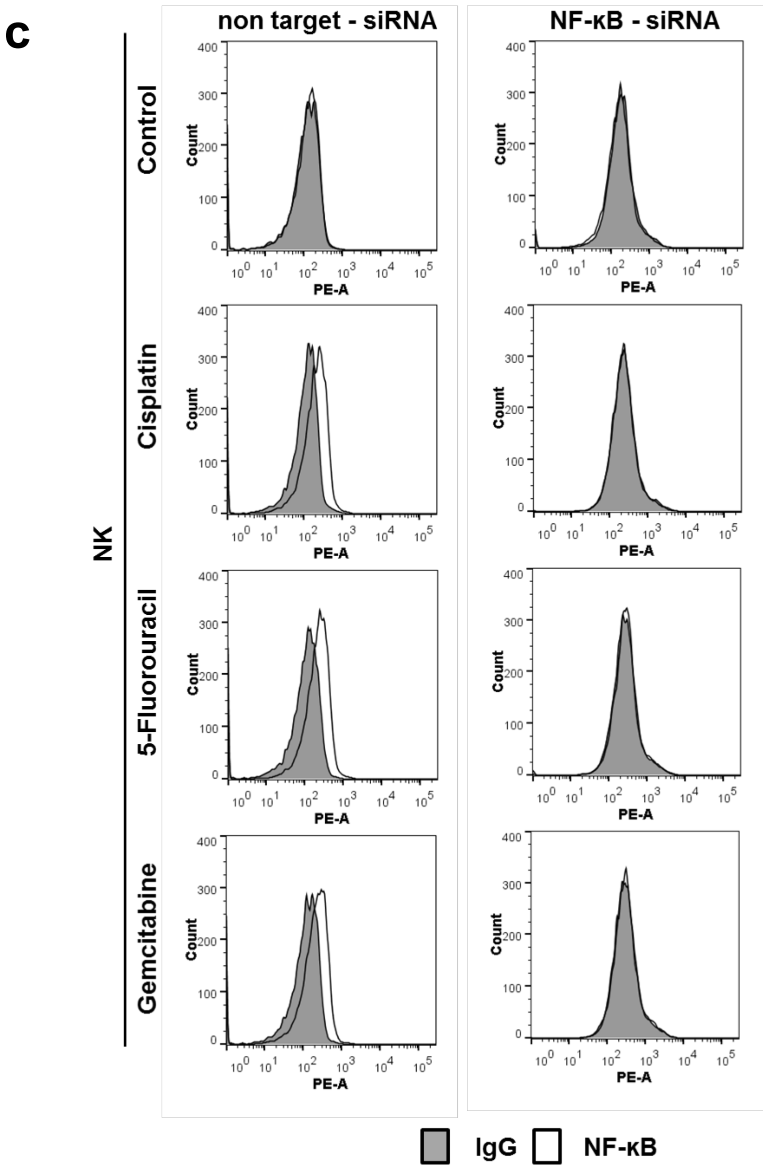
c
non target - siRNA
NF-κB - siRNA
NK
Control
Cisplatin
5-Fluorouracil
Gemcitabine
Count
PE-A
IgG
NF-κB

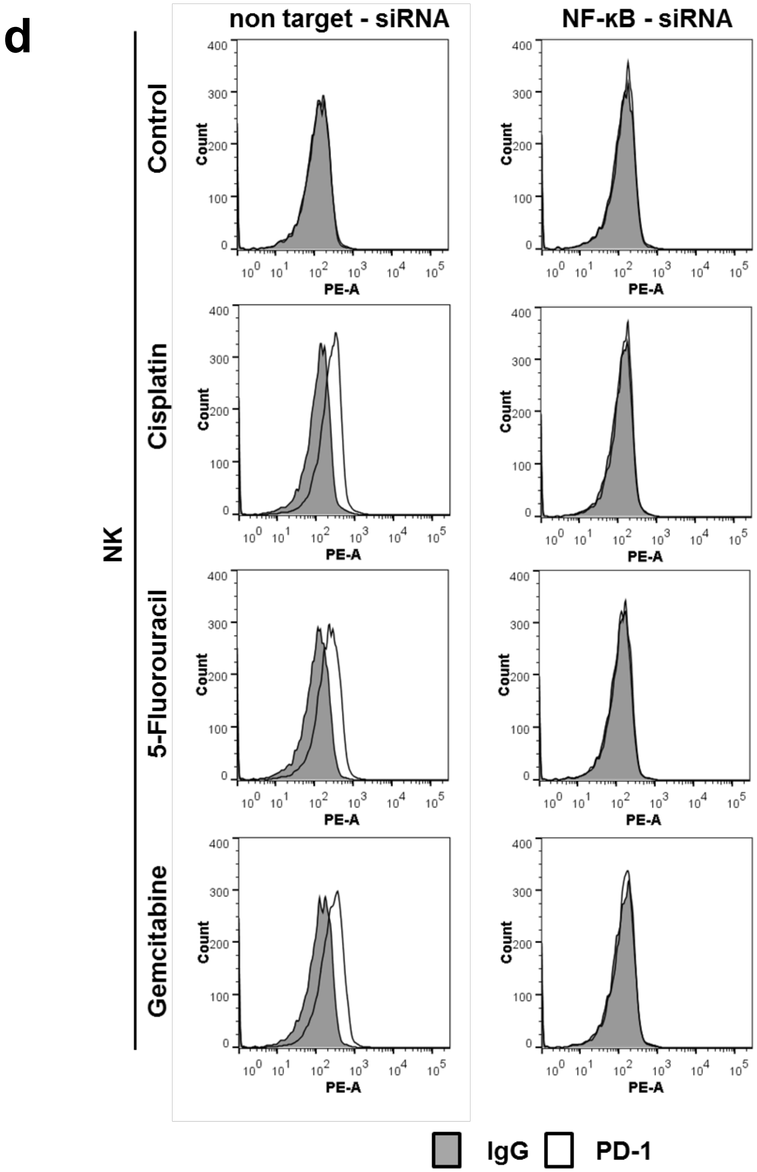
d
non target - siRNA
NF-κB - siRNA
NK
Control
Cisplatin
5-Fluorouracil
Gemcitabine
Count
PE-A
IgG
PD-1
